# Development and Validation of a LC-QTOF-MS/MS Method to Assess the Phenolic Profile of Pulse Flours

**DOI:** 10.3390/molecules30132730

**Published:** 2025-06-25

**Authors:** Achilleas Panagiotis Zalidis, Natasa P. Kalogiouri, Ioannis Mourtzinos, Dimitris Sarris, Konstantinos Gkatzionis

**Affiliations:** 1 Laboratory of Consumer and Sensory Perception of Food & Drinks, Department of Food Science and Nutrition, School of the Environment, University of the Aegean, Metropolite Ioakeim 2, Myrina, 81400 Lemnos, Greece; fnsd21005@aegean.gr (A.P.Z.); dsarris@aegean.gr (D.S.); 2 Laboratory of Analytical Chemistry, Department of Physical, Analytical and Environmental Chemistry, School of Chemistry, Aristotle University of Thessaloniki, 54124 Thessaloniki, Greece; 3 Laboratory of Food Chemistry and Biochemistry, Department of Food Science and Technology, School of Agriculture, Aristotle University of Thessaloniki, 54124 Thessaloniki, Greece; mourtzinos@auth.gr

**Keywords:** bioactive compounds, phenolics, liquid chromatography, functional flours, pulse flours, lupin flour, chickpea flour

## Abstract

Functional flours, defined as flours enriched with health-promoting compounds such as phenolics, fibers, or proteins, are gaining attention as wheat-free alternatives due to the nutritional limitations of wheat flour. This study introduces a novel liquid chromatographic time-of-flight tandem mass spectrometric method (LC-QTOF-MS/MS) to characterize the phenolic profiles of functional flours from different origins and evaluate their potential as flour substitutes in food products. The proposed method was validated and the limits of quantification (LOQs) were calculated over the ranges 0.1–1.0 mg/kg. Calculated recoveries were as low as 82.4%. Repeatability and reproducibility were expressed as intra-day (n = 6) and inter-day (n = 4 × 3) measurements and were lower than 8.1 and 10.9%, respectively. Target and suspect screening findings underscore the potential of pulse flours as nutritionally enriched ingredients for functional food development.

## 1. Introduction

The world estimated wheat production (including wheat flour in wheat equivalent) would reach 770.8 million tons by the end of 2023 [1]. However, in recent years, there has been a slow and steady increase in consumer interest in wheat-free foods, driven in part by an increasing awareness of celiac disease [2] and by the poor nutritional properties of wheat flour [3]. These factors have dictated the rise of functional foods and flours that could represent good options for product enrichment including pulses, barley, and polyphenol-rich by products. Functional foods are described as foods fortified with special constituents that possess advantageous physiological effects and serve multiple functions [4]. Most commonly, the identified functions of functional foods include nutritional function, health benefits, and the technological process during their development [5]. The functional properties are attributed based on the complex linkage between composition, the molecular conformation of a structure, and the physicochemical characteristics of food components [6]. The functional properties of flours are primarily influenced by the components of food material as well as the structure of these components [7]. The key components in flours are protein, soluble and insoluble fibers, resistant starch, and certain bioactive ingredients. In recent years, studies have been focused on the phenolic compounds present in different flours due to their antihypertensive and antibacterial actions, antioxidant activity, and potential health benefits related to their protective effects on oxidative stress [8]. However, different authors have reported that common gluten-free foods are of poor nutritional quality when compared with their gluten-containing counterparts [9]. To increase the nutritional value of GF products, the addition of raw materials of different origins is gaining interest [10]. Pulses are a category of leguminous crops that includes beans, lentils, chickpeas, and peas. They are a good source of complex carbohydrates and rich in dietary fibre, protein, vitamins, minerals, and polyphenols [11]. Additionally, they could support plant-based diets which tend to lack ‘complete’ proteins [12]. Lupin has been previously associated with health-promoting benefits and contains substantial quantities of phenolic acids including protocatechuic, vanillic, p-coumaric, and ferulic acid among others [13]. Furthermore, recent analyses discovered the presence of apigenin derivatives and isoflavones (e.g., genistein). Chickpeas are highly appraised for their nutritional benefits. Chickpeas contain significant amounts of isoflavones (daidzein and genistein), while flavonols such as quercetin, kaempferol, and myricetin have also been identified [14].

Pulse flours deriving from the milling of legumes can be utilized as value-added ingredients for several cereal-based food products ranging from baking goods to pasta and snacks [15]. The processing treatments for flour preparation are critical for the chemical composition and nutritional properties of the milled product [16]. Despite growing interest, comprehensive studies focusing on the quantification of the bioactive compounds in pulse flours remain limited. A reduction in the phenolics in pulse flours due to heat treatment has been previously established, while kaempferol-O-hexoside was the most abundant compound in yellow beans [17]. Furthermore, phenolic profiling has been performed in prosopis pod flour via High Resolution Mass Spectrometry (HRMS)highlighting the potential use of the flour as a functional food while identifying eight anthocyanins and 13 phenolic compounds [18]. Chickpea flour has been analyzed by deploying HRMS and certain phenolic compounds, such as flavonoids, phenolic acids, and lignans, were identified as valuable sources of health-promoting compounds [10].

Based on the above, incorporating pulse flours in foods could boost their nutritional benefits along with their health-promoting properties. HRMS has proved its excellent analytical performance by allowing the analysis of a wide range of compounds in food [19]. Therefore, it could be used for the characterization of the flours, providing screening and tentative identification for both target and non-target compounds. The use of liquid chromatography time-of-flight tandem mass spectrometry (LC-QTOF-MS/MS) enables the identification of unknown analytes with high accuracy and could be effectively applied in the analysis and characterization of the phenolic compounds in milled powders [20,21].

The aim of this study was to develop and validate an analytical method using LC-QTOF-MS/MS for the quantification of bioactive compounds in various pulse flours. This method is intended to provide quantitative indicators that can support the development of nutritionally enriched and functional food products. To this end, flours derived from wheat, lupins, and chickpeas were analyzed. The validated LC-QTOF-MS/MS method was further enhanced by the integration of comprehensive suspect screening, enabling the detection of a wider range of phenolic compounds, including several not previously reported in flour matrices.

## 2. Results and Discussion

### 2.1. Method Validation Results

The validation results are presented in Table S2 in the Supplementary Material. The calibration curves presented good linearity for all the analytes over the range LOQ- 10 mg/kg (R^2^ values (0.9921–0.9995). LOQs and LODs were calculated over the ranges 0.1–1.0 mg/kg and 0.03–0.2 mg/kg, respectively. Τhe accuracy of the LC-QTOF-MS/MS method was evaluated, and the calculated recoveries were as low as 82.4%. Repeatability and reproducibility results were expressed as intra-day (n = 6) and inter-day (n = 4 × 3) measurements and were calculated to be lower than 8.1 and 10.9%, respectively. No significant matrix effect (ME%) was observed.

### 2.2. Target Screening Results

By scanning the flour samples and with the initial target list (Table S2, Supplementary Material) in the reference, the presence of 16 target compounds was determined (Table 1). These compounds included apigenin, caffeic acid, catechin, coumaric acid, diosmin, epicatechin, epigallocatechin, ferulic acid, epicatechin gallate, kaempferol, luteolin, myricetin, myricitrin, naringenin, quercetin, quercitrin, protocatechuic acid, rutin, sinapic acid, taxifolin, vanillic acid, and vanillin. Their presence was verified after comparing the experimental molecular ions and retention time with the matching standards. A maximum threshold of ΔRt = 0.2 was set. The most abundant fragments provided by the MS/MS spectra were recorded along with their elemental formula and the compounds were quantified using the peak areas. The target screening results for each matrix are summarized in Table 1. The EICs, MS, and MS/MS spectra are presented in Figures S1–S27 in the Supplementary Material.

Considering the phenolic compounds in wheat, apigenin, coumaric acid, chrysin, diosmin, ferulic acid, naringin, rutin, and vanillin were detected and have been previously identified in the literature [22,23,24,25]. Among the detected compounds, ferulic acid was detected in WFL and WFL. Recent studies have highlighted its strong antioxidant properties, as well as its applications in both the food and pharmaceutical industries [26]. Additionally, vanillin, a phenolic compound recognized for its anti-inflammatory properties and widespread use in the nutraceutical industry [27], was also present in higher concentrations in WFL (0.53 mg/kg, SD = 0.06) compared to WFC (0.40 mg/kg, SD = 0.06). Coumaric acid, a natural antioxidant which has been shown to decrease low density lipoprotein (LDL) peroxidation and the risk of stomach cancer, was also present in both WFC and WFL. Conversely, apigenin was detected in trace amounts in both flours while diosmin and naringin were at similar concentrations in both WFC and WFL, suggesting that their levels may not be significantly influenced by flour type or geographical origin in this context. However, rutin, which is considered to belong among the top therapeutically active phytochemicals [28], was detected only in WFC. These variations in concentration could be attributed to multiple factors, including milling methods, agricultural practices, climatic conditions, and cultivar differences [22]. Overall, the concentrations of phenolic compounds in the tested wheat flours were significantly lower than those reported in previous studies [29]. This difference could potentially be attributed to the removal of wheat bran and germ during the processing of WFC and WFL, as these components are rich in antioxidants [30].

Pulse flours exhibited a greater diversity of detected phenolic constituents, with lupin flours (LFC and LFL) showing elevated concentrations compared to wheat flour, aligning with findings in the literature [31]. The identified compounds included apigenin, caffeic acid, coumaric acid, diosmin, ferulic acid, kaempferol, quercetin, taxifolin, vanillin, and vanillic acid. Some of these phenolic compounds, such as caffeic acid, kaempferol, and quercetin, are known to be indigenous to pulses [32]. As shown in Table 1, ferulic acid was identified as the most abundant phenolic compound in lupin flour, with similar concentrations in both the origins of 1.67 mg/kg (SD = 0.02) in LFC and 1.58 mg/kg (SD = 0.07) in LFL, aligning with previous reports on pulses [33]. Both LFC and LFL reported the detection of kaempferol, with the literature pointing to a positive association between the consumption of foods containing kaempferol and a reduced risk of developing several disorders such as cancer and cardiovascular diseases [34]. Vanillic acid, a dihydroxybenzoic acid analog, was also higher in LFC, where it has been utilized as a flavoring agent in foods and cosmetics with effective neuroprotective properties [35].

**Figure 1:**
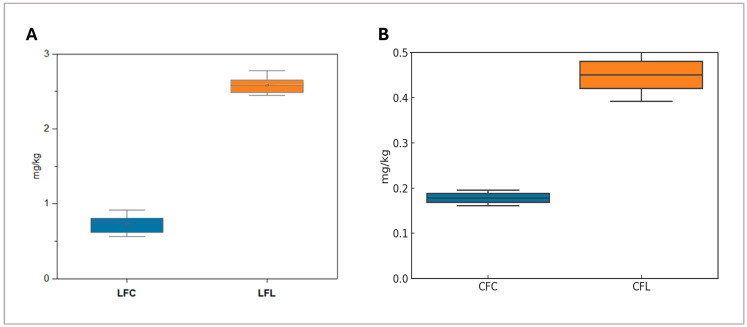
(**A**) Vanillin concentration in commercial lupin flour (LFC) and lupin flour from Lemnos (LFL), highlighting the potential role of geographical or processing differences; (**B**) quercitrin levels in chickpea flour from Lemnos (CFL) compared to commercial chickpea flour (CFC), illustrating the potential role of source location or processing differences.

In contrast, taxifolin was detected exclusively in LFL with prior studies highlighting its protective effects against cardiovascular diseases, inflammation, and viral infections [36]. Additionally, apigenin, a dietary flavonoid with documented nutraceutical benefits [37,38], was present in both lupin flours. Vanillin levels were also significantly higher (*p* = 0.0003) in LFL (2.5 mg/kg, SD = 0.1) compared to LFC (0.724 mg/kg, SD = 0.11), which underlines the impact of geographical origin and processing on phenolic composition, as illustrated in Figure 1A.

Similar to lupin flours, chickpea flours exhibited a broader range of phenolic compounds than wheat flours which included apigenin, coumaric acid, chrysin, diosmin, ferulic acid, kaempferol, luteolin, naringin, quercetin, rutin, and sinapic acid, which are shown in Table 1. Diosmin was the most abundant phenolic compound, a flavone glycoside whose health benefits have been validated in numerous in vitro and in vivo studies and include antioxidative, antihyperglycemic, anti-inflammatory, antimutagenic, and antiulcer properties [39]. Its concentration was notably higher in CFC (0.72 mg/kg, SD = 0.03). Moreover, both CFC and CFL contained key bioactive compounds such as apigenin, coumaric acid, kaempferol, and naringin. Notably, quercitrin—a compound known to positively influence gut microbiota composition, thereby enhancing overall gut health [40] —demonstrated significantly higher levels (*p* = 0.0002) in CFL compared to CFC (Figure 1B). Additionally, sinapic acid, recognized for its strong antioxidant properties [41], was also more abundant in CFL. Comparing the two different origins of chickpeas flours, it was observed that CFL displayed a more robust and well-balanced phenolic profile, with higher concentrations of key bioactive compounds. This suggests its potential application as a functional food with enhanced health benefits. The findings from target screening highlight the superior phenolic profile of both commercial and Lemnos pulse flours compared to wheat flours. Lupin and chickpea flours exhibited valuable bioactive compounds which are linked with numerous antioxidant, anti-inflammatory, and anti-cancer properties. Commercially available flours offer higher concentrations of specific phenolic compounds (i.e., kaempferol in LFC and diosmin in CFC) which might be useful for specific applications in the food and health industry. However, the Lemnos varieties may offer a broader range of compounds with higher concentrations, enhancing their potential for functional food applications.

To further interpret the variability observed in phenolic compound concentrations, a granulometric analysis was performed on all the flour samples. The results indicated that LFC and CFC contained a higher proportion of fine particles below 250 µm. In contrast, LFL and CFL exhibited a broader particle size distribution, with a substantial proportion retained at 500–600 µm. These differences are likely owed to the use of different milling methods—industrial roller milling for the commercial flours versus traditional stone milling for the Lemnos flours. Coarser particle sizes may limit solvent penetration during extraction and partially explain the variation in measured phenolic content.

### 2.3. Suspect Screening Results

The flour samples were further screened using the suspect screening lists (Tables S2–S4, Supplementary Material) created from the literature. The presence of the compounds was tentatively verified on the basis of the accurate mass and examining the MS/MS spectra using in silico fragmentation tolls such as MetFrag and literature records. In Table 1, Table 2 and Table 3, the suspect screening results for each matrix are presented, providing information about the MS/MS fragmentation of each compound and the experimental tR. The suspect compounds were tentatively semi-quantified using the calibration curves of same-class compound derivatives. In total, 18 compounds were identified in wheat flour, 29 in lupin flour, and 34 in chickpea flour.

The suspect screening of WFC and WFL, detailed in Table 2, identified a range of phytochemical compounds, encompassing phenolic acids, flavonoids, and stilbenes, specifically the following: 2,4-dihydroxybenzoic acid, 4-hydroxybenzoic acid, Apigenin-6-C-arabinoside-8-C-hexoside, Apigenin-7-O-neohesperidoside, Formononetin, Isovitexin-2″-O-rhamnoside, Kaempferol-7-O-sopheroside, Methylisoorientin-2″-O-rhamnoside, Nobiletin, Quercetin-3-O-rutinose, Salicylic acid, Tricin, and Vicenin-2 (apigenin-6,8-di-C-glucoside).

For both origins, the most abundant phenolic compounds belong to the class of phenolic acids including three hydroxybenzoic acids and a ferulic acid derivative, such as 2,4-dihydroxybenzoic acid which is present in both WFC and WFL, similar to the findings of Hernandez et al. [22]. Hydroxybenzoic acids, as the primary aromatic secondary metabolites, lend distinct organoleptic characteristics to food [42] and are associated with numerous health benefits [43]. Additionally, studies have shown that the advancement of hydroxybenzoic acids in functional foods holds promise in alleviating common ailments including inflammation, nervous system disorders, and cerebrovascular or cardiovascular conditions [44]. Furthermore, formononentin, an isoflavone, was detected in wheat flours and has been associated with the prevention and treatment of several diseases [45]. Overall, WFL exhibited a lower concentration of the detected phenolic compounds compared to WFC, which could be attributed to the absence of wheat bran and germ during the milling process.

The suspect screening for lupin flours is presented in Table 3, which identified a variety of phytochemicals, including isochromans, flavonoids, terpenoids, phenolic acids, and secoiridoids: 2′-Hydroxygenistein 7-O-glucoside, 2′-hydroxygenistein, Abscisic acid, Apigenin-7-O-β-glucopyranoside, Apiin, Baicalein, Chlorogenic acid, Chrysoeriol, Cichoriin, Dicaffeoylquinic acid, Eriodictyol, Genistein, Genistein 8-C-glucosidee 2, Genistein C-diglucoside 3, Licodione, Luteolin 7-O-glucoside, Luteolin-4′-O-glucoside, Luteone, Vicenin 2, and Vitexin.

Chrysoeriol, genistein, and genistein derivatives were detected solely in LFC [46]. Additionally, isochromans, which are recognized for their applications in pharmacological practices, were notably more abundant in LFC compared to LFL [47]. Similar, baicalein, a promising bioactive flavone with anti-cancer properties, was in higher concentration in LFC [48]. In comparison, LFL exhibited high concentrations of dicaffeoylquinic acid, eriodictyol, and vicenin 2, compounds previously reported by Zhong et al. [49]. Vicenin 2, abundant in LFL, has been linked to hepatoprotective properties and holds potential for developing therapeutic strategies targeting various aspects of diabetes [50]. Eriodictyol, a compound previously reported in germinated lupin seeds [51], was detected in both lupin flours. Similar, dicaffeoylquinic acid was also identified in both LFC and LFL. Furthermore, flavonoids were present including licodione, luteone, vitexin, and wighteone. These results are in agreement with Ranilla et al. [52], who previously identified isoflavones in lupin cultivars, and Das et al. [53], who highlighted the health benefits of isoflavones in plants of the Fabaceae family.

In Table 4, the suspect screening results for the chickpea flours are shown. The identified compounds include (Epi)afzelechin, Apigenin-6-C-glucoside, Benzoic acid, Biochanin A 7-O-β-D-glucopyranoside, Daidzein, Dihydroxybenzoic acid hexoside, Dihydroxybenzoic acid malonyl hexoside, Dihydroxybenzoic acid pentoside, Gallic acid hexoside, Genistein, Isorhamentin 3-O-β-D-glucopyranoside, Kaempferol 3-O-rutinoside, Malvidin, Methyl isoflavone isomer I, Myricetin-3-O-rhamnoside, Naringenin, Orobol, p-hydroxybenzoic acid, Pratensein/Kaemferide, Prunin [naringenin 7-O-β-D-glucopyranoside], Quercetin-3-O-galactoside, and Quercetin-3-O-rhamnoside.

The major phenolic classes identified in the chickpea flours were hydroxybenzoic acids and derivatives and flavonoids [54,55]. Specifically, CFC displayed higher concentrations of hydroxybenzoic acids compared to CFL, namely p-hydroxybenzoic acid, gallic acid, Dihydroxybenzoic acid hexoside, Dihydroxybenzoic acid hexoside deoxyhexoside, and Dihydroxybenzoic acid malonyl hexoside. Hydroxybenzoic acids have been previously studied for their biological properties, and it is well documented that their use in the food industry enhances the nutritional profile of foods [56,57]. Additionally, malvidin, an anthocyanin with reported antioxidant and anticancer properties [58], was detected in the chickpea flours along with biochanin B and a methyl isoflavone isomer, indicating a strong antioxidant profile. Furthermore, orobol, a genistein derivative with pharmacological and nutritional properties, was exclusively detected in CFL [59].

The suspect screening analysis of pulse flours highlighted distinct compositional differences between the commercial pulse flours (LFC and CFC) and the Lemnos variety (LFL and CFL), emphasizing the impact of genetic and processing factors on their phytochemical profiles. Compared to wheat flour, which exhibited a lower diversity and concentration of bioactive compounds, both the lupin and chickpea flours demonstrated a significantly richer phytochemical composition, reinforcing their potential as superior functional ingredients for health-promoting food applications. These findings underscore the functional differentiation between commercial and Lemnos variety pulse flours, with LFC and CFC aligning more with general nutritional enhancement, whereas LFL and CFL exhibit profiles rich in specialized bioactives, which could potentially act as biomarkers and offer targeted health benefits and origin distinction.

This study provides valuable insights, but it is important to acknowledge certain limitations. The sample size (n = 10 per flour type), though representative, is relatively modest and drawn from a single geographic location (Lemnos, Greece). This specificity might limit a broader application of the results. Additionally, despite observing minimal matrix effects during method validation, their potential influence within complex flour matrices should be noted. Future research should aim to include a wider array of geographic sources and processing conditions to enhance the applicability of these findings.

## 3. Materials and Methods

### 3.1. Flour Samples

Thirty flours were sourced locally from Lemnos, Greece and included ten wheat flour (WFL) samples, ten lupin flour (LFL) samples, and ten chickpea flour (CFL) samples. Sample preparation was as follows: Regarding WFL, the seed is threshed, a stage in which its bran is removed and then ground in a mill and grinding was carried out in a cooperating mill. LFL and CFL were ground in a small-scale stone mill until a fine powder was obtained. The commercially available flours corresponding to the aforementioned flours were also procured. Additionally, the ten commercial wheat flours (WFC) were purchased from the Mills of Saint George (Sourpi, Greece), the ten commercial chickpea flours (CFC) were purchased from Bioagros (Pella, Greece), and the ten commercial lupin flours (LFC) were purchased from Lup’Ingredients (Martigne-Ferchaud, France).

### 3.2. Granulometric Analysis

To assess the particle size distribution of the flour samples and account for potential differences arising from the milling processes, a granulometric analysis was performed using a vibratory sieve shaker (Retax, Labor Siebmaschine, Type LS10, Nr 4082, Hemmingen, Germany) based on the standard AACC method 66-20.01 [60]. Approximately 100 g of each flour sample were sieved through a standardized stack of stainless-steel sieves with mesh sizes of 600, 500, 350, 250, 175, 79.5, and 9 µm for a duration of 15 min at a constant amplitude. The retained weight after each sieve was recorded, and the results were expressed as a percentage of the total sample weight (Table S1, Supplementary Material). This analysis was conducted for both the commercial and Lemnos flours.

### 3.3. Chemicals and Standards

Methanol and water (LC-MS grade) were purchased from HiPerSolv CHROMANORM, VWR Chemicals BDH (Amsterdam, The Netherlands). Formic acid 98–100% was purchased from Merck (Darmstadt, Germany). For the determination of phenolic compounds, apigenin 98%, caffeic acid 98%, p-coumaric acid 97%, chrysin 97%, ferulic acid 98%, diosmin 97%, kaempferol 98%, luteolin 98%, naringenin 98%, quercetin 98%, quercitrin 99%, rutin 98%, sinapic acid 98%, taxifolin 98%, vanillic acid 98%, vanillin 98%, and hesperidin 98% (internal standard), were purchased from Sigma-Aldrich (Stenheim, Germany).

### 3.4. Preparation of Standard Solutions

Standard stock solutions of all the phenolic compounds were weighted and diluted in LC-MS grade methanol to a final concentration of 1000 mg/L. All the stock standard solutions were stored at −20 °C in dark brown glass bottles to prevent photodegradation. Working solutions were prepared by further dilution of the stock solution using methanol–water (80:20, *v*/*v*).

### 3.5. Sample Preparation

For sample preparation, 0.1 g of a flour sample was weighed in an Eppendorf tube and 1 mL of methanol–water (80:20, *v*/*v*) was added for the extraction of bioactive compounds. There was no incubation applied to the samples. The mixture was vortexed for 1 min and then centrifuged at 14,000 rpm at 25 °C for 10 min. Then, the extract was collected and filtered using 0.22 μm nylon syringe filters (Captiva, Agilent Technologies, Santa Clara, CA, USA). Hesperidin was added as an internal standard at a 1 mg/kg concentration level to monitor instrument response, and the samples were then directly injected into the chromatographic system.

### 3.6. Method Validation

Method validation was implemented to ensure the suitability of the proposed LC-QTOF-MS/MS method by evaluating linearity, the limits of detection (LOD), and the limits of quantification (LOQ), accuracy, reproducibility, matrix effect (ME%), repeatability, and reproducibility. The calibration curves were constructed using the standard addition method over the range 0.1, 0.25, 0.5, 1, 2.5, and 5mg/kg in methanol:water (80:20, *v*/*v*). The accuracy of the method was evaluated by calculating the %RE of the analytes at three concentration levels (LOQ, 1 mg/kg, and 5 mg/kg), depending on the linear range of the standard addition curve of each amino acid. As for the limit of quantification (LOQ), the lowest point of each calibration curve that corresponded to a signal-to-noise ratio > 10 was set. The limit of detection (LOD) was calculated by dividing the LOQ by 3.3 (signal-to-noise ratio of 3). For the estimation of %ME, one concentration level was considered (5 mg/kg); this was obtained by dividing the analyte’s response in a post-extraction spiked sample by its response in the standard solution, and then subtracting 1 from the result. Positive values indicate signal enhancement, while negative values indicate signal suppression. Repeatability (intra-day precision) and reproducibility (inter-day precision) were expressed as the %RSD values of the three concentration levels, with six replicates analyzed on the same day and three replicates analyzed over three consecutive days (3 × 3), respectively.

### 3.7. Instrumental Analysis

Chromatographic analysis was carried out using a using an ExionAC LC system (SCIEX, Framingham, MA, USA) interfaced to a quadrupole Time-of-Flight (QTOF) mass spectrometer Sciex Exion LC/QTOF X500R, SCIEX, Framingham, MA, USA). The eluted compounds were introduced into the mass spectrometer via an ESI turbo VTM source operated in the negative ion mode. TOF–MS and TOF–MS/MS data were acquired using the information-dependent acquisition (IDA) electrospray ionization mode. Nebulizer gas was set at 55 psi, heater gas at 50 psi, and curtain gas at 30 psi. The Ion-spray voltage was 4500 V, and the de-clustering potential was 80 V. The MS/MS spectra were acquired using a collision energy of 45 eV with a collision energy spread of 15 eV. External calibration was performed prior to analysis using a cluster solution provided by SCIEX, while internal calibration was conducted by injecting the calibration solution at the start of each run and once every five samples during batch acquisition. Mass spectra were recorded within an *m/z* range of 50–1000, with an accumulation time of 0.25 s. MS/MS experiments were carried out in the data-dependent acquisition mode, targeting the 10 most abundant precursor ions per full scan, with an accumulation time of 0.08 s. Sample acquisition was managed using SCIEX OS software (v3.4.5), which was also used to generate the extracted ion chromatograms. The applied parameters included a mass accuracy window of 5 ppm, an S/N threshold of 3, a minimum area threshold of 1000, and a minimum intensity threshold of 500.

The chromatographic separation was carried out with a C18 column (2.1 × 100 mm, 2.6 µm) from Fortis (Cheshire, UK) thermostated at 40 °C. Mobile phase (A) consisted of 90% water, 10% methanol with 0.1% formic acid and mobile phase (B) of 100% methanol with 0.1% formic acid. For the analysis of phenolic compounds, the gradient elution started with 1% of organic phase (B) (flow rate 0.2 mL min^−1^) for 1 min, gradually increasing to 39% for the next 4 min, and then increasing to 95% (12–15 min) and remaining constant for the following 3 min (flow rate 0.4 mL min^−1^). Then, the organic phase increased gradually to 99% at a flow rate of 0.2 mL min^−1^, within 1 min and remained constant for another 4 min (16–20 min). Finally, the system was returned to its initial conditions (1% B–99% A), which were restored within 0.1 min (flow rate decreased to 0.2 mL min^−1^) to re-equilibrate the column for 5 min prior to the next injection.

### 3.8. Screening Methodology

#### 3.8.1. Target Screening

A target list was created that included ten significant phenolic acids that have been identified in plant-based foods, eighteen flavonoids found in plants, seeds, and roots, and two methoxyphenols. The target list is presented in Table S2 in the Supplementary Material. The classification of the compounds was made using FoodDB [61]. For every target compound, extracted ion chromatograms (EICs) of the precursor ions were made and evaluated throughout the samples using the Analytics package of the SCIEX OS software. The target compounds included in the list were screened in the samples according to the following parameters that were set: mass accuracy of the precursor ion and the MS/MS fragments with a selection window of 5 ppm, a retention time tolerance of (tR < 0.2 min), a response peak area threshold of above 1000, and a peak intensity of at least 800.

#### 3.8.2. Suspect Screening

In suspect screening, a suspect database was generated from the literature, including all the phenolic compounds that have previously been identified independently in wheat, lupins, and chickpeas, in order to scan the samples for their presence. The in-house suspect database consisted of the following: 55 phenolic compounds found in wheat plants and seeds; 74 compounds found in lupin plants and seeds; and 98 compounds found in chickpea plants. The suspect lists are shown at Tables S4–S6 in the Supplementary Material and the classification of the compounds was made using FoodDB. In the case of a peak being detected in the matrix, the presence of the suspect compound was determined by a careful analysis of the MS/MS fragments and a comparison with those in the mass spectral libraries, or by in silico fragmentation with tools like MetFrag [62] and MassBank [63]. MetFrag was employed using the neutral exact mass (with a mass error of 5 ppm) and the appropriate ionization mode while MassBank used the compound name, exact mass (tolerance = 0.3), and molecular formula in the negative ionization mode.

### 3.9. Statistical Analysis

The experiments were performed using 10 samples from each flour, and the mean and standard deviation of the values were calculated. Statistical analysis was performed with IBM SPSS Statistics v.23 software (Armonk, NY, USA: IBM Corp), namely the differences among the values were determined using a one-way analysis of variance (ANOVA). ANOVA was followed by post hoc Tukey’s HSD tests to determine the pairwise differences between flour types. Differences were considered statistically significant at *p* < 0.05.

## 4. Conclusions

The wheat flours exhibited significantly lower phenolic concentrations compared to the pulse flours, likely due to the removal of bran and germ during processing. Ferulic acid was the dominant compound in the wheat flours, with WFL containing marginally higher levels, while other compounds like rutin and vanillin were detected only in specific samples and at lower concentrations. The lupin and chickpea flours displayed a wider and more abundant range of phenolic compounds. LFC and LFL both contained ferulic acid and kaempferol, but LFL was particularly rich in vanillin. The chickpea flours also demonstrated high compositional variability with CFC, showcasing higher diosmin concentrations, whereas CFL showed higher levels of quercitrin and sinapic acid. These variations reflect the combined influence of botanical origin and geographical factors.

Suspect screening confirmed the presence of additional bioactives—such as hydroxybenzoic acids, flavonoid glycosides, and isoflavones—many of which were unique to specific flour types or origins. The variations found between the commercial and Lemnos variety flours suggest the significant impact of geographical factors, e.g., soil composition and climate, on the phenolic fingerprint of such matrices. Certain compounds identified show promise as biomarkers for flour authenticity and quality assessment.

In conclusion, the integration of targeted and suspect screening strategies enables a more comprehensive investigation of phenolic compounds, facilitating the identification of both the expected and novel bioactive molecules. By employing these approaches, discriminative markers could be identified to distinguish different flour types, authenticate their origin, and enhance their application in functional food production. Future research could focus on tailoring processing and agricultural practices for producers and manufacturers to harness the full potential of pulse-based ingredients for the creation of foods with targeted health benefits. This combination of advanced screening methods and the optimization of flour production could provide a pathway for developing more nutritionally enriched, health-oriented, and functional food products.

## Figures and Tables

**Table 1:** Target screening results for commercial flours (WFC, LFC, CFC) and flours from Lemnos, Greece (WFL, LFL, CFL). Statistical significance is denoted by asterisks based on Tukey’s HSD test and differences were considered statistically significant at *p* < 0.05.

Compound	Molecular Formula	[M − H]^−^ Standard	[M − H]^−^ Experimental CFC	[M − H]^−^ Experimental CFL	Rt (min)	ΔRt	Fragments *m/z*	Elemental Formula	WFC (SD) mg/kg	WFL (SD) mg/kg	LFC (SD) mg/kg	LFL (SD) mg/kg	CFC (SD) mg/kg	CFL (SD) mg/kg
**Apigenin**	C_15_H_10_O_5_	269.0448	269.0451	269.0451	9.04	+0.02	63.0238 65.0027 107.0133 117.0335 149.0237 159.0448 225.0553	[C_5_H_4_]-H^−^ [C_4_H_3_O-H]-H^−^ [C_6_H_4_O_2_]-H^−^ [C_8_H_6_O]-H^−^ [C_8_H_5_O_3_]^−^ [C_10_H_7_O_2_]^−^ [C_14_H_9_O_3_]^−^		**<LOQ**	**<LOQ**	**<LOQ**	**<LOQ**	**<LOQ**
**Caffeic acid**	C_9_H_8_O_4_	179.0348	179.0351	179.0355	5.12	+0.06	90.9983 134.0323 134.9878 135.0456	[C_4_H_2_O]-H^−^ [C_6_H_5_+2H]^−^ [C_8_H_7_O_2_]-H^−^ [C_8_H_7_O_2_]^−^			**<LOQ**	**<LOQ**		
***p*-Coumaric acid**	C_9_H_8_O_3_	163.0400	163.0401	163.0402	5.97	−0.01	65.0389 93.0344 117.0343 119.0501	[C_5_H_4_+H]^−^ [C_6_H_5_O]^−^ [C_8_H_7_O-H]-H^−^ [C_8_H_7_O]^−^	**<LOQ**	**<LOQ**	**<LOQ**	**<LOQ**	**<LOQ**	**<LOQ**
**Chrysin**	C_15_H_10_O_4_	253.0510	253.0509	253.0507	10.54	−0.01	63.0238 107.0146 143.0494 209.0609	[C_5_H_5_-H]-H^−^ [C_6_H_4_O_2_]-H^−^ [C_10_H_7_O]^−^ [C_14_H_9_O_2_]^−^						
**Diosmin**	C_28_H_32_O_15_	607.1669	607.1674	607.1671	7.17	+0.08	151.0397 199.0616 283.0266 284.0331 443.0990	[C_8_H_7_O_3_]^−^ [C_9_H_12_O_5_]-H^−^ [C_15_H_8_O_6_]-H^−^ [C_15_H_8_O_6_]^−^ [C_22_H_21_O_10_-H]-H^−^	**<LOQ**	**<LOQ**	**<LOQ**	**<LOQ**	0.72 * **(0.04)**	**<LOQ**
**Ferulic acid**	C_10_H_10_O_4_	193.0505	193.0509	193.0507	6.25	−0.03	106.0414 132.0210 133.0301 134.0375 178.0276	[C_7_H_6_O]^−^ [C_8_H_6_O_2_-H]-H^−^ [C_8_H_6_O_2_]-H^−^ [C_8_H_6_O_2_]^−^ [C_9_H_7_O_4_]-H^−^	**<LOQ**	**<LOQ**	1.67 ** **(0.24)**	1.58 ** **(0.08)**	**<LOQ**	**<LOQ**
**Kaempferol**	C_15_H_10_O_6_	285.0398	285.0403	285.0410	8.92	0	65.0030 93.0343 117.0344 151.0037 211.0406 229.0509	[C_4_H_3_O-H]-H^−^ [C_6_H_5_O]^−^ [C_8_H_5_O]^−^ [C_7_H_4_O_4_]-H^−^ [C_13_H_8_O_3_]-H^−^ [C_13_H_9_O_4_]^−^			**<LOQ**	**<LOQ**	**<LOQ**	**<LOQ**
**Luteolin**	C_15_H_10_O-	285.0401	285.0402	285.0410	8.36	0	65.0036 107.0138 132.0221 151.0038 199.0402 217.0515 241.0504	[C_4_H_3_O-H]-H^−^ [C_6_H_5_O_2_-H]-H^−^ [C_8_H_6_O_2_-H]-H^−^ [C_7_H_4_O_4_]-H^−^ [C_12_H_6_O_3_+H]^−^ [C_12_H_9_O_4_]^−^ [C_14_H_9_O_4_]^−^					**<LOQ**	**<LOQ**
**Naringin**	C_27_H_32_O_14_	579.1714	579.1716	579.1715	6.78	−0.15	119.0495 151.0035 271.0615 313.0709	[C_8_H_8_O]-H^−^ [C_7_H_3_O_4_]^−^ [C_15_H_11_O_5_]^−^ [C_17_H_13_O_6_]^−^	**<LOQ**	**<LOQ**			**<LOQ**	**<LOQ**
**Quercetin**	C_15_H_10_O_7_	301.0350	301.0422	301.0363	8.08	0	65.0029 83.0137 107.0135 121.0292 151.0034 178.9982 186.0319	[C_4_H_3_O-H]-H^−^ [C_4_H_4_O_2_]-H^−^ [C_6_H_5_O_2_-H]-H^−^ [C_7_H_5_O_2_]^−^ [C_7_H_4_O_4_]-H^−^ [C_8_H_5_O_5_-H]-H^−^ [C_11_H_7_O_3_]-H^−^			**<LOQ**	**<LOQ**		
**Quercitrin**	C_21_H_20_O_11_	447.0930	447.0943	447.0935	7.19	0.05	151.0040 255.0301 284.0326 285.0409 300.0282 301.0367 327.0529	[C_7_H_4_O_4_]-H^−^ [C_14_H_9_O_5_-H]-H^−^ [C_15_H_9_O_6_]-H^−^ [C_15_H_9_O_6_]^−^ [C_15_H_9_O_7_]-H^−^ [C_15_H_9_O_7_]^−^ [C_17_H_11_O_7_]^−^					0.18 * **(0.02)**	0.45 * **(0.06)**
**Rutin**	C_27_H_30_O_16_	609.1456	609.1462	609.1472	6.67	+0.05	151.0041 255.0300 271.0250 300.0266 301.0350	[C_7_H_4_O_4_]-H^−^ [C_14_H_9_O_5_-H]-H^−^ [C_14_H_9_O_6_-H]-H^−^ [C_15_H_9_O_7_]-H^−^ [C_15_H_9_O_7_]^−^	**<LOQ**	**ND**			**<LOQ**	**<LOQ**
**Sinapic acid**	C_11_H_12_O_5_	223.0611	223.0614	223.0615	6.26	−0.02	67.0188 68.9981 69.0345 93.0351 121.0307 127.0412 149.0253 181.0492 193.0159	[C_4_H_4_O]-H^−^ [C_3_H_3_O_2_-H]-H^−^ [C_4_H_4_O+H]^−^ [C_6_H_6_O]-H^−^ [C_7_H_6_O_2_]-H^−^ [C_6_H_8_O_3_]-H^−^ [C_8_H_7_O_3_-H]-H^−^ [C_9_H_6_O_5_]-H^−^					**<LOQ**	0.52 * **(0.05)**
**Taxifolin**	C_15_H_12_O_7_	ND	303.0603	303.0518	6.09	−0.01	57.0339 83.0142 121.0294 123.0450 125.0244 175.0409 217.0504 285.0417	[C_3_H_3_O+2H]^−^ [C_4_H_4_O_2_]-H^−^ [C_7_H_6_O_2_]-H^−^ [C_7_H_6_O_2_+H]^−^ [C_6_H_4_O_3_+H]^−^ [C_10_H_7_O_3_]^−^ [C_12_H_8_O_4_+H]^−^ [C_15_H_11_O_6_-H]-H^−^			**ND**	**<LOQ**		
**Vanillin**	C_8_H_8_O_3_	151.0400	151.0403	151.0404	5.64	−0.015	92.0268 108.0214 136.0165	[C_6_H_4_O]^−^ [C_6_H_4_O_2_]^−^ [C_7_H_5_O_3_]-H^−^	0.40 * **(0.06)**	0.54 * **(0.07)**	0.72 * **(0.11)**	2.58 * **(0.11)**		
**Vanillic acid**	C_8_H_8_O_4_	167.0351	167.0350	167.0351	5.13	0	65.0034 91.0189 108.0217 123.0094 124.0167 152.0117	[C_4_H_2_O]-H^−^ [C_6_H_4_O]-H^−^ [C_6_H_4_O_2_]^−^ [C_6_H_5_O_3_-H]-H^−^ [C_6_H_5_O_3_]-H^−^ [C_7_H_5_O_4_]-H^−^			0.98 * **(0.09)**	**<LOQ**		

ND: not detected * *p* < 0.05. ** *p* > 0.05.

**Table 2:** Suspect screening results for commercial wheat flour (WFC) and wheat flour from Lemnos (WFL).

Compound	Molecular Formula	[M − H]^−^ Experimental	Rt (min)	Fragments *m/z*	Elemental Formula	Mass Bank ID
**2,4-dihydroxybenzoic acid**	C_7_H_6_O_4_	153.0195	5.28	67.0187 91.0200 109.0286 135.0088	[C_4_H_4_O]-H^−^ [C_6_H_4_O]-H^−^ [C_6_H_5_O_2_]^−^ [C_7_H_5_O_3_-H]-H^−^	BS003106
**4-hydroxybenzoic acid**	C_7_H_6_O_3_	137.0245	7.32	65.0397 93.0345	[C_5_H_4_+H]^−^ [C_6_H_5_O]^−^	PR100596
**Apigenin-6-C-arabinoside-8-C-hexoside**	C_26_H_28_O_14_	563.1412	6.00	191.0345 283.0610 383.0764 563.1384		PR309300
**Apigenin-7-O-neohesperidoside**	C_27_H_30_O_14_	577.1568	6.98	269.0077 269.0464	[C_14_H_8_O_6_-2H]-H^−^ [C_15_H_9_O_5_]^−^	PR305867
**Formononetin**	C_16_H_12_O_4_	443.1416	13.16	125.0243 195.0458	[C_6_H_3_O_3_+2H]^−^ [C_13_H_7_O_2_]^−^	BS003359
**Isovitexin-2″-O-rhamnoside**	C_27_H_30_O_14_	577.1580	6.98	125.0261 269.0465 577.2257	[C_15_H_9_O_5_]^−^	PR100821
**Kaempferol-7-O-sopheroside**	C_27_H_30_O_16_	609.1813	6.95	125.0246 164.0117 286.0491 301.0707	[C_6_H_3_O_3_+2H]^−^ [C_8_H_4_O_4_]^−^ [C_15_H_9_O_6_+H]^−^ [C_16_H_13_O_6_]^−^	PR307127
**Methylisoorientin-2″-O-rhamnoside**	C_28_H_32_O_15_	607.1681	7.11	255.0322 284.0382 299.0571 607.1674	[C_16_H_10_O_6_+H]^−^	
**Nobiletin**	C_21_H_22_O_8_	401.1200	11.83	175.1088 307.1011 313.0678 373.1295	[C_20_H_22_O_7_]-H^−^	MoNA_0001836
**Quercetin-3-O-rutinose**	C_27_H_30_O_16_	609.1828	6.95	125.0246 242.0591 286.0491 609.1822	[C_6_H_4_O_3_+H]^−^ [C_14_H_10_O_4_]^−^ [C_15_H_9_O_6_+H]^−^	PR309250
**Salicylic acid**	C_12_H_16_O_3_	ND	10.01	109.0285 122.0389 150.0364 207.1025	[C_6_H_5_O_2_]^−^ [C_7_H_5_O_2_+H]^−^	BS003127
**Tricin**	C_17_H_14_O_7_	329.0677	9.10	151.0032 161.0250 227.0346 243.0296 299.0214	[C_7_H_4_O_4_]-H^−^ [C_9_H_4_O_3_+H]^−^ [C_13_H_7_O_4_]^−^ [C_13_H_9_O_5_-H]-H^−^ [C_15_H_8_O_7_]-H^−^	FIO00747
**Vicenin-2 (apigenin-6,8-di-C-glucoside)**	C_27_H_30_O_15_	593.1527	6.16	312.0661 383.0799 413.0885 473.1109 503.1191	[C_17_H_11_O_6_+H]^−^ [C_20_H_16_O_8_]-H^−^ [C_21_H_18_O_9_]-H^−^ [C_23_H_22_O_11_]-H^−^ [C_24_H_24_O_12_]-H^−^	PR309303

ND: not detected.

**Table 3:** Suspect screening results for commercial lupin flour (LFC) and lupin flour from Lemnos (LFL).

Compound	Molecular Formula	[M − H]^−^ experimental LFC	Rt (min)	Fragments *m/z*	Elemental Formula	Mass Bank ID
**2′-Hydroxygenistein 7-O-glucoside**	C_21_H_21_O_11_	448.0975	6.37	284.1642 285.0941 413.8154 414.8164		PN000117
**2′-hydroxygenistein**	C_15_H_10_O_6_	285.0406	7.60	65.0033 133.0295 175.0403 199.0405 217.0509	[C_4_H_4_O_2_]-H^−^ [C_8_H_6_O_2_]-H^−^ [C_10_H_7_O_3_]^−^ [C_12_H_6_O_3_+H]^−^ [C_12_H_9_O_4_]^−^	PN000005
**Abscisic acid**	C_15_H_20_O_4_	263.1290	7.44	136.0533 189.0918 188.0841 204.1157 219.1382	[C_8_H_10_O_2_-H]-H^−^ [C_12_H_15_O_2_-2H]-H^−^ [C_12_H_15_O_2_-H]-H^−^ [C_13_H_16_O_2_]^−^ [C_14_H_19_O_2_]^−^	BML00506
**Apigenin 4′, 7-O-diglucoside**	C_27_H_31_O_15_	594.1553	5.58	353.0676 354.0706 473.1129 504.1241	[C_19_H_15_O_7_-H]-H^−^ [C_23_H_22_O_11_]^−^ [C_24_H_24_O_12_]^−^	BS003711
**Apiin**	C_26_H_28_O_14_	563.1412	6.54	59.0134 293.0461 311.0562 341.0674 413.0886	[C_2_H_4_O_2_]-H^−^ [C_17_H_11_O_5_-H]-H^−^ [C_17_H_11_O_6_]^−^ [C_18_H_14_O_7_]-H^−^ [C_21_H_19_O_9_-H]-H^−^	BS003825
**Baicalein**	C_15_H_10_O_5_	269.0454	8.54	63.0239 132.0216 133.0294 159.0452	[C_5_H_5_-H]-H^−^ [C_8_H_5_O_2_]-H^−^ [C_8_H_5_O_2_]^−^ [C_10_H_6_O_2_+H]^−^	PR307464
**Chlorogenic acid**	C_16_H_18_O_9_	353.1020	10.78	133.0293 219.0667 285.1136 353.1024	[C_8_H_7_O_2_-H]-H^−^ [C_12_H_11_O_4_]^−^	FIO00627
**Chrysoeriol**	C_16_H_12_O_6_	299.0566	8.67	87.0088 227.0346 255.0282 284.0324	[C_13_H_8_O_4_]-H^−^ [C_14_H_9_O_5_-H]-H^−^ [C_15_H_9_O_6_]-H^−^	BS003344
**Cichoriin**	C_15_H_16_O_10_	355.0602	0.88	70.9983 178.0479 180.0636 224.0531	[C_6_H_11_O_6_]-H^−^ [C_6_H_11_O_6_+H]^−^	
**Dicaffeoylquinic acid**	C_25_H_24_O_12_	515.1259	1.06	87.0088 111.0082 154.9988 515.1265	[C_3_H_3_O_3_]^−^ [C_5_H_5_O_3_-H]-H^−^ [C_6_H_6_O_5_-2H]-H^−^	PR309023
**Eriodictyol**	C_15_H_12_O_6_	287.0562	6.77	57.0344 65.0033 83.0137 125.0243 152.0117 177.0559	[C_3_H_3_O+2H]^−^ [C_4_H_3_O-H]-H^−^ [C_4_H_4_O_2_]-H^−^ [C_6_H_4_O_3_+H]^−^ [C_7_H_4_O_4_]^−^ [C_10_H_9_O_3_]^−^	PR309310
**Genistein**	C_15_H_10_O_5_	269.0454	8.54	63.0239 107.0136 132.0216 133.0294 159.0452	[C_5_H_4_]-H^−^ [C_6_H_4_O_2_]-H^−^ [C_8_H_6_O_2_-H]-H^−^ [C_8_H_6_O_2_]-H^−^ [C_10_H_7_O_2_]^−^	PR305516
**Genistein 6-C-glucoside 1**	C_21_H_21_O_10_	432.1085	4.01	99.0563 254.0790 272.0893 306.0770	[C_12_H_13_O_6_+H]^−^ [C_12_H_14_O_7_+2H]^−^ [C_15_H_12_O_7_+2H]^−^	PN000015
**Genistein C-diglucoside 3**	C_27_H_31_O_15_	594.1553	5.57	353.0676 473.1129 474.1138	[C_19_H_16_O_7_-2H]-H^−^ [C_23_H_23_O_11_]-H^−^	PN000054
**Licodione**	C_15_H_12_O_5_	271.0702	4.13	119.0506 142.0669 159.0941 203.0833	[C_8_H_6_O+H]^−^	PR310754
**Luteolin 7-O-glucoside**	C_21_H_20_O_11_	447.0936	6.36	59.0140 151.0039 217.0518 284.0336 285.0409	[C_2_H_4_O_2_]-H^−^ [C_7_H_3_O_4_]^−^ [C_12_H_8_O_4_+H]^−^ [C_15_H_9_O_6_]-H^−^ [C_15_H_9_O_6_]^−^	PR305631
**Luteolin-4′-O-glucoside**	C_21_H_20_O_11_	447.0936	6.36	59.0140 151.0039 217.0518 284.0336 285.0409	[C_2_H_4_O_2_]-H^−^ [C_7_H_3_O_4_]^−^ [C_12_H_8_O_4_+H]^−^ [C_15_H_9_O_6_]-H^−^ [C_15_H_9_O_6_]^−^	PR305690
**Vicenin 2**	C_27_H_30_O_15_	593.1509	5.56	323.0567 353.0658 383.0767 473.1083 503.1198	[C_18_H_12_O_6_]-H^−^ [C_19_H_14_O_7_]-H^−^ [C_20_H_16_O_8_]-H^−^ [C_23_H_22_O_11_]-H^−^ [C_24_H_24_O_12_]-H^−^	PR309303
**Vitexin**	C_21_H_20_O_10_	431.0985	6.89	211.0400 239.0355 268.0376 269.0452 267.0309	[C_13_H_7_O_3_]^−^ [C_14_H_9_O_4_-H]-H^−^ [C_15_H_9_O_5_-H]-H^−^[C_15_H_9_O_5_]-H^−^ [C_15_H_9_O_5_]^−^	FIO00915

**Table 4:** Suspect screening results for commercial chickpea flour (CFC) and chickpea flour from Lemnos (CFL).

Compound	Molecular Formula	[M − H]^−^ Experimental CFC	Rt (min)	Fragments *m/z*	Elemental Formula	Mass Bank ID
**(Epi)afzelechin**	C_15_H_14_O_5_	273.0769	7.02	65.0393 93.0349 109.0304 137.0250 165.0198	[C_5_H_4_+H]^−^ [C_6_H_5_O]^−^ [C_6_H_4_O_2_+H]^−^ [C_7_H_6_O_3_]-H^−^ [C_8_H_7_O_4_-H]-H^−^	QTOF007573
**Apigenin-6-C-glucoside**	C_21_H_20_O_10_	431.1181	3.64	93.0332 137.0227 299.0761	[C_6_H_5_O]^−^ [C_13_H_14_O_8_+H]^−^	PR302849
**Benzoic acid**	C_7_H_6_O_2_	121.0294	6.59	77.0391 93.0338	[C_6_H_5_]^−^ [C_6_H_4_O+H]^−^	KO000320
**Biochanin A 7-O-β-D-glucopyranoside**	C_22_H_22_O_10_	445.1148	8.47	165.0202 267.0316 268.0383 283.0613	[C_8_H_6_O_4_]-H^−^ [C_15_H_8_O_5_]-H^−^ [C_15_H_8_O_5_]^−^ [C_16_H_11_O_5_]^−^	PR302874
**Biochanin B**	C_16_H_12_O_4_	267.0665	9.633.2	91.0181 132.0219 195.0452 223.0403 252.0426	[C_6_H_4_O]-H^−^ [C_8_H_5_O_2_]-H^−^ [C_13_H_7_O_2_]^−^ [C_14_H_9_O_3_-H]-H^−^ [C_15_H_9_O_4_]-H^−^	BS003040
**Daidzein**	C_15_H_10_O_4_	253.0509	10.53	63.0243 145.0308 209.0609	[C_5_H_4_]-H^−^ [C_9_H_6_O_2_]-H^−^ [C_14_H_9_O_2_]^−^	PR309180
**Gallic acid hexoside**	C_13_H_16_O_10_	331.0675	2.26	123.0086 124.0163 125.0244 149.9959 168.0066 313.0575	[C_6_H_5_O_3_-H]-H^−^ [C_6_H_5_O_3_]-H^−^ [C_6_H_5_O_3_]^−^ [C_7_H_4_O_4_-H]-H^−^ [C_7_H_5_O_5_]-H^−^ [C_13_H_15_O_9_-H]-H^−^	PR309053
**Genistein**	C_15_H_10_O_5_	269.0451	9.06	63.0239 107.0136 132.0216 133.0294 159.0452	[C_5_H_4_]-H^−^ [C_6_H_4_O_2_]-H^−^ [C_8_H_6_O_2_-H]-H^−^ [C_8_H_6_O_2_]-H^−^ [C_10_H_7_O_2_]^−^	PR305516
**Kaempferol 3-O-rutinoside**	C_27_H_30_O_15_	593.1521	7.21	285.0418 284.0339 533.3009	[C_15_H_9_O_6_]-H^−^ [C_15_H_9_O_6_]^−^	PR306656
**Malvidin**	C_17_H_15_O_7_	330.0741	9.93	96.0212 139.1129 172.1062 212.1365	[C_5_H_5_O_2_]-H^−^	PR020010
**Myricetin-3-O-rhamnoside**	C_21_H_20_O_12_	463.0891	6.71	151.0038 271.0250 301.0362 300.0289	[C_7_H_4_O_4_]-H^−^ [C_14_H_9_O_6_-H]-H^−^ [C_15_H_9_O_7_]-H^−^ [C_15_H_9_O_7_]^−^	PT209290
**Naringenin**	C_15_H_12_O_5_	271.0706	4.02	74.0246 116.0504 142.0662 159.0928 203.0825 225.0660		PR309309
**p-hydroxybenzoic acid**	C_7_H_6_O_3_	137.0243	2.30	65.0394 93.0343	[C_5_H_4_+H]^−^ [C_6_H_5_O]^−^	R100596
**Pratensein/Kaemferide**	C_16_H_12_O_6_	299.0566	8.81	107.0128 148.0161 227.0354 255.0311 284.0327	[C_6_H_4_O_2_]-H^−^ [C_8_H_5_O_3_]-H^−^ [C_13_H_8_O_4_]-H^−^ [C_14_H_9_O_5_-H]-H^−^ [C_15_H_9_O_6_]-H^−^	BML01860
**Prunin [naringenin 7-O-β-D-glucopyranoside]**	C_21_H_22_O_10_	433.1262	3.66	93.0346 137.0246 263.0729	[C_6_H_5_O]^−^ [C_7_H_3_O_3_+2H]^−^	PR040149
**Quercetin-3-O-galactoside**	C_21_H_20_O_12_	463.0886	6.69	179.0000 255.0308 271.0243 300.0280 301.0359	[C_8_H_4_O_5_]-H^−^ [C_14_H_9_O_5_-H]-H^−^ [C_14_H_9_O_6_-H]-H^−^ [C_15_H_9_O_7_]-H^−^ [C_15_H_9_O_7_]^−^	PR309229
**Quercetin-3-O-rhamnoside**	C_21_H_20_O_11_	447.1140	4.18	108.0214 152.0107 163.0399 315.0725 447.1125	[C_6_H_5_O_2_]-H^−^ [C_7_H_4_O_4_]^−^ [C_9_H_5_O_3_+2H]^−^ [C_13_H_14_O_9_+H]^−^	PR305653

## Data Availability

Data are available upon request.

## Supplementary Materials

The following supporting information can be downloaded at https://www.mdpi.com/article/10.3390/molecules30132730/s1: Table S1: Granulometric composition of wheat, lupin, and chickpea flours from commercial and Lemnos sources.; Table S2: Validation results. Table S3: Target screening list of compounds; Table S4: Suspect screening list for wheat flour; Table S5: Suspect screening list for lupin flour; Table S6: Suspect screening list for chickpea flour; Figure S1: Extracted ion chromatogram, MS, and MS/MS spectra of taxifolin in LFL; Figure S2: Extracted ion chromatogram, MS, and MS/MS spectra of vanillic acid in LFC; Figure S3: Extracted ion chromatogram, MS, and MS/MS spectra of vanillin in LFL; Figure S4: Extracted ion chromatogram, MS, and MS/MS spectra of vanillin in WFL; Figure S5: Extracted ion chromatogram, MS, and MS/MS spectra of apigenin in LFL; Figure S6: Extracted ion chromatogram, MS, and MS/MS spectra of caffeic acid in LFL; Figure S7: Extracted ion chromatogram, MS, and MS/MS spectra of chrysin in CFL; Figure S8: Extracted ion chromatogram, MS, and MS/MS spectra of chrysin in LFC; Figure S9: Extracted ion chromatogram, MS, and MS/MS spectra of chrysin in WFL; Figure S10: Extracted ion chromatogram, MS, and MS/MS spectra of coumaric acid in CFL; Figure S11: Extracted ion chromatogram, MS, and MS/MS spectra of coumaric acid in WFL; Figure S12: Extracted ion chromatogram, MS, and MS/MS spectra of diosmin in CFL; Figure S13: Extracted ion chromatogram, MS, and MS/MS spectra of diosmin in WFL; Figure S14: Extracted ion chromatogram, MS, and MS/MS spectra of ferulic acid in CFL; Figure S15: Extracted ion chromatogram, MS, and MS/MS spectra of ferulic acid in LFC; Figure S16: Extracted ion chromatogram, MS, and MS/MS spectra of ferulic acid in WFL; Figure S17: Extracted ion chromatogram, MS, and MS/MS spectra of kaempferol in CFL; Figure S18: Extracted ion chromatogram, MS, and MS/MS spectra of kaempferol in CFC; Figure S19: Extracted ion chromatogram, MS, and MS/MS spectra of kaempferol in LFL; Figure S20: Extracted ion chromatogram, MS, and MS/MS spectra of luteolin in CFL; Figure S21: Extracted ion chromatogram, MS, and MS/MS spectra of luteolin in LFL; Figure S22: Extracted ion chromatogram, MS, and MS/MS spectra of naringin in WFC; Figure S23: Extracted ion chromatogram, MS, and MS/MS spectra of naringin in CFL; Figure S24: Extracted ion chromatogram, MS, and MS/MS spectra of protocatechuic acid in CFL; Figure S25: Extracted ion chromatogram, MS, and MS/MS spectra of quercitrin in CFL; Figure S26: Extracted ion chromatogram, MS, and MS/MS spectra of rutin in CFL; and Figure S27: Extracted ion chromatogram, MS, and MS/MS spectra of rutin in WFC.
