# Supplementary material for: Development and Validation of a LC-QTOF-MS/MS Method to Assess the Phenolic Profile of Pulse Flours
Source: Molecules. 2025 Jun 25;30(13):2730. doi: 10.3390/molecules30132730 (PMC12251335; doi:10.3390/molecules30132730)
Supplement: Supplementary file 1 [file molecules-30-02730-s001.zip › molecules-3694306-supplementary.pdf]

Supplementary material

Development and Validation of a LC-QTOF-MS/MS Method to Assess the Phenolic Profile of Pulse Flours

Achilleas Panagiotis Zalidis [1], Natasa P. Kalogiouri [2,*], Ioannis Mourtzinos [3], Dimitris Sarris [1] and Konstantinos Gkatzionis [1,*]

[1] Laboratory of Consumer and Sensory Perception of Food & Drinks, Department of Food Science and
Nutrition, School of the Environment, University of the Aegean, Metropolite Ioakeim 2, Myrina,
81400 Lemnos, Greece; fnsd21005@aegean.gr (A.P.Z.); dsarris@aegean.gr (D.S.)

[2] Laboratory of Analytical Chemistry, Department of Physical, Analytical and Environmental Chemistry, School of Chemistry, Aristotle University of Thessaloniki, 54124 Thessaloniki, Greece

[3] Laboratory of Food Chemistry and Biochemistry, Department of Food Science and Technology, School of Agriculture, Aristotle University of Thessaloniki, 54124 Thessaloniki, Greece; mourtzinos@auth.gr

* Correspondence: kalogiourin@chem.auth.gr (N.P.K.); kgkatzionis@aegean.gr (K.G.)

Table S1. Granulometric composition of wheat, lupin, and chickpea flours from commercial and Lemnos sources.

Mean particle size (µm)	WFC (%)	WFL (%)	LFC (%)	LFL (%)	CFC (%)	CFL (%)	GSFC (%)	GSFL (%)	OSFC (%)	OSFL (%)
**9**	6.49	0.27	0.37	0.32	0.35	0.48	0.30	0.32	0.23	0.36
**79.5**	18.66	55.56	4.86	1.47	1.20	3.74	1.98	0.96	0.36	3.32
**175**	4.43	11.37	2.16	3.82	1.87	2.39	1.52	1.15	0.68	1.69
**250**	21.08	27.54	6.79	6.97	2.38	5.64	6.75	5.07	2.18	2.54
**350**	1.14	2.97	10.85	13.76	4.46	3.02	2.21	4.67	10.55	11.55
**500**	0.68	1.86	36.72	24.27	16.24	54.24	20.73	48.38	75.03	51.73
**600**	47.52	0.44	38.26	49.39	73.50	30.49	66.50	39.46	10.98	28.81

***WFC: wheat flour commercial; WFL: wheat flour Lemnos; LFC: lupin flour commercial; LFL: lupin flour Lemnos; CFC: chickpea flour commercial; CFL: chickpea flour Lemnos; GSFC: grape seed flour commercial; GSFL: grape seed flour Lemnos; OSFC: olive stone flour commercial; OSFL: olive stone flour Lemnos**

Table S2. Validation results

Compound	Equation $y = (a \pm Sa) + (b \pm Sb)x$ Linearity range: LOQ – 5 mg/Kg	R^2	RE %	LOD (mg/Kg)	LOQ (mg/Kg)	Intra-day % RSD (n = 6) Concentration Level (mg/Kg)			Inter-day % RSD (n = 3 × 3) Concentration Level (mg/Kg)			% Matrix Effect
						LOQ	1	5	LOQ	1	5	
Apigenin	y = (55655224 ± 32522) x + (212649 ± 102221)	0.9932	88.1	0.2	0.5	4.3	4.2	5.0	3.1	4.1	4.2	-9.1
Caffeic acid	y = (4689561 ± 15522) x + (135224 ± 30255)	0.9925	82.4	0.2	0.8	2.1	3.3	2.7	3.5	3.1	4.1	-8.5
p-Coumaric acid	y = (2565452 ± 100224) x - (7950 ± 6552)	0.9921	90.5	0.2	0.5	3.4	8.1	2.5	5.2	3.8	4.3	-10.6
Chrysin	y = (95221 ± 4235) x + (50321 ± 10214)	0.9935	83.4	0.2	0.5	2.1	3.5	4.7	4.4	6.4	3.5	-12.5
Diosmin	y = (1865458 ± 19252) x + (215423 ± 53452)	0.9995	92.7	0.2	0.5	3.2	2.9	2.6	2.9	5.1	3.2	-7.5
Ferulic acid	y = (1745523 ± 6325) x + (61563 ± 15654)	0.9992	89.1	0.3	1.0	1.4	2.7	5.9	3.4	3.7	4.0	-9.9
Kaempferol	y = (178562 ± 10121) x + (32565 ± 18452)	0.9971	88.7	0.3	0.9	2.2	2.1	5.1	5.4	5.8	3.2	-12.2
Luteolin	y = (10565853 ± 124563) x + (365452 ± 304523)	0.9985	86.3	0.03	0.1	3.4	2.5	4.6	3.2	3.7	5.1	-6.5
Naringin	y = (22532241 ± 1235232) x + (12252324 ± 3020456)	0.9932	92.4	0.2	0.8	2.3	1.8	1.9	4.1	2.4	5.0	-10.4
Quercetin	y = (11452568 ± 554562) x + (5032652 ± 1452565)	0.9944	93.3	0.2	0.8	2.4	1.3	2.1	5.2	1.2	4.1	-8.8
Quercitrin	y = (2452326 ± 22352) x - (165251 ± 50451)	0.9985	92.4	0.03	0.1	2.5	1.2	2.1	4.7	3.5	10.9	-11.4
Rutin	y = (16525844 ± 545625) x + (3565245 ± 1315425)	0.9966	95.4	0.2	0.7	1.6	1.9	3.3	4.3	2.8	4.6	-5.6
Sinapic acid	y = (10645287 ± 9154630) x - (355456 ± 221145)	0.9974	93.4	0.2	0.5	2.4	1.4	2.2	4.1	4.1	5.1	-9.5
Taxifolin	y = (9456544 ± 235006) x + (940632 ± 523248)	0.9984	92.7	0.2	0.5	2.7	2.5	2.3	4.4	5.1	4.1	-8.2
Vanillin	y = (821065 ± 2224) x + (27556 ± 5652)	0.9965	91.1	0.03	0.1	1.5	3.7	3.4	4.2	4.1	4.4	-11.5
Vanillic acid	y = (435651 ± 4108) x - (10454 ± 10335)	0.9978	90.8	0.3	0.9	4.3	2.6	2.5	3.5	3.2	4.2	-14.7

Table S3. Target screening list of compounds

Compound	Molecular formula	Class
**Apigenin**	$C_{15}H_{10}O_5$	Flavonoids
**Caffeic acid**	$C_9H_8O_4$	Cinnamic acids and derivatives
**Catechin**	$C_{15}H_{14}O_6$	Flavonoids
**Chrysin**	$C_{15}H_{10}O_4$	Flavonoids
**Cinammic acid**	$C_{14}H_{10}N_2O_4S$	Cinnamic acids and derivatives
**Coumaric acid**	$C_9H_8O_3$	Cinnamic acids and derivatives
**Diosmin**	$C_{28}H_{32}O_{15}$	Flavonoids
**Epicatechin**	$C_{15}H_{14}O_6$	Flavonoids
**Epigallocatechin**	$C_{15}H_{14}O_7$	Flavonoids
**Ferulic acid**	$C_{10}H_{10}O_4$	Hydroxycinnamic acid
**Gallic acid**	$C_7H_6O_5$	Benzene and substituted derivatives
**Epicatechin gallate**	$C_{22}H_{18}O_{10}$	Flavonoids
**Hesperidin**	$C_{28}H_{34}O_{15}$	Flavonoids
**Kaempferol**	$C_{15}H_{10}O_6$	Flavonoids
**Luteolin**	$C_{15}H_{10}O_6$	Flavonoids
**Myricetin**	$C_{15}H_{10}O_8$	Flavonoids
**Myricitrin**	$C_{21}H_{20}O_{12}$	Flavonoids
**Naringin**	$C_{27}H_{32}O_{14}$	Flavonoids
**Quercetin**	$C_{15}H_{10}O_7$	Flavonoids
**Quercitrin**	$C_{21}H_{20}O_{11}$	Flavonoids
**Rosmarinic acid**	$C_{18}H_{16}O_8$	Cinnamic acids and derivatives
**Protocatechuic acid**	$C_7H_6O_4$	hydroxybenzoic acid derivatives
**Rutin**	$C_{27}H_{30}O_{16}$	Flavonoids
**Sinapic acid**	$C_{11}H_{12}O_5$	Hydroxycinnamic acids
**Syringaldehyde**	$C_9H_{10}O_4$	Phenols
**Syringic acid**	$C_9H_{10}O_5$	Benzene and substituted derivatives
**Taxifolin**	$C_{15}H_{12}O_7$	Flavonoids
**Vanillic acid**	$C_8H_8O_4$	Benzene and substituted derivatives
**Vanillin**	$C_8H_8O_3$	Phenols

Table S4. Suspect screening list for wheat flour

Compound	Molecular formula	Class
**2,4-Dihydroxybenzoic acid**	$C_7H_6O_4$	Phenolic acids
**3′,4′,5′-trihydroxy-3,7-dimethylflavone**	$C_{17}H_{14}O_7$	Flavonoids
**4-Hydroxybenzoic acid**	$C_7H_6O_3$	Benzene and substituted derivatives
**Cyanidin**	$C_{15}H_{11}O_6Cl$	Flavonoids
**Apigenin-6-C-arabinoside-8-C-hexoside**	$C_{26}H_{28}O_{14}$	Flavonoids (8-c-glycosides)
**apigenin-7-O-neohesperidoside**	$C_{27}H_{30}O_{14}$	Flavonoids (O-glycosides)
**Chlorogenic acid**	$C_{16}H_{18}O_9$	Phenolic acid
**chrysoeriol-7-O-neosperidoside**	$C_{28}H_{32}O_{15}$	Flavonoids (O-glycosides)
**Coumarin**	$C_9H_6O_2$	Coumarins
**Cyanidin-3-glucoside**	$C_{21}H_{21}O_{11}$	Flavonoids
**Cyanidin-3-glucoside (kuromanin)**	$C_{21}H_{21}O_{11}Cl$	Flavonoids
**Delphinidin-3-glucoside**	$C_{21}H_{21}O_{12}$	Hydroxyflavonoids
**Dihydroferulic acid**	$C_{20}H_{18}O_8$	Phenylpropanoic acids
**Ellagic acid**	$C_{14}H_6O_8$	Tannins
**Formononetin**	$C_{23}H_{24}O_9$	Isoflavonoids
**Hinokinin**	$C_{20}H_{18}O_6$	Lignan
**Hydrocinnamic acid**	$C_9H_{10}O_2$	Phenylpropanoids
**Hydroxytyrosol**	$C_8H_{10}O_3$	Phenolic alcohols
**Isovitexin-2″-O-rhamnoside**	$C_{27}H_{30}O_{14}$	Flavonoids
**kaempferol-7-O-neohesperidoside**	$C_{27}H_{30}O_{15}$	Flavonoids (O-glycosides)
**kaempferol-7-O-sopheroside**	$C_{27}H_{30}O_{16}$	Flavonoids (O-glycosides)
**Lucenin-1/3 (luteolin-6/8-C-xyloside-8/6-C-glucoside)**	$C_{26}H_{28}O_{15}$	Flavonoids (8-c-glycosides)
**luteolin-7-O-neohesperidoside**	$C_{27}H_{30}O_{15}$	Flavonoids (O-glycosides)
**Methylisoorientin-2″-O-rhamnoside**	$C_{28}H_{32}O_{15}$	Flavonoids
**Nobiletin**	$C_{21}H_{22}O_8$	Flavonoids
**Pelargonidin-3-glucoside (callistephin)**	$C_{21}H_{21}O_{10}Cl$	Flavonoids
**Phloridzin**	$C_{21}H_{24}O_{10}$	Flavonoids
**Pinoresinol**	$C_{20}H_{22}O_6$	Lignan
**Pinosylvin**	$C_{26}H_{32}O_{12}$	Stilbenes
**Pinosylvin (double glycosylation)**	$C_{26}H_{32}O_{12}$	Stilbenes

**Procatechuic acid**	$C_7H_6O_4$	Phenolic acids
**Procyanidin B3**	$C_{30}H_{26}O_{12}$	Proanthocyanidins
**Prodelphinidin B3**	$C_{30}H_{26}O_{14}$	Proanthocyanidins
**Prunin**	$C_{21}H_{22}O_{10}$	Flavonoids
**quercetin-3-O-rutinose**	$C_{27}H_{30}O_{16}$	Flavonoids (O-glycosides)
**Salicylic acid**	$C_{12}H_{16}O_3$	Benzene and substituted derivatives
**trans-caffeic acid**	$C_9H_8O_4$	Phenolic acids
**trans-p-coumaric acid**	$C_9H_8O_3$	Phenolic acids
**Tricin**	$C_{17}H_{14}O_7$	Flavonoids
**Vicenin-2 (apigenin-6,8-di-C-glucoside)**	$C_{27}H_{30}O_{15}$	Flavonoids (8-c-glycosides)
**Vitexin/isovitexin**	$C_{21}H_{20}O_{10}$	Flavonoids

Table S5. Suspect screening list for lupin flour

**Compound**	**Molecular formula**	**Class**
**1-(3'-methoxy-4'-hydroxy)-phenyl-6,7-dihydroxyisochroman**	$C_{16}H_{16}O_5$	Benzopyrans
**1-phenyl-6,7-dihydroxy-isochroman**	$C_{15}H_{14}O_3$	Benzopyrans
**2′-Hydroxygenistein 7-O-glucoside**	$C_{21}H_{21}O_{11}$	Flavonoids (O-glycosides)
**2′-Hydroxygenistein O-glucoside malonylated (I)**	$C_{24}H_{23}O_{14}$	Flavonoids (O-glycosides)
**Kaempferol-glucoside 3**	$C_{22}H_{23}O_{10}$	Flavonoids
**2'-hydroxygenistein**	$C_{15}H_{10}O_6$	Flavonoids
**4-Hydroxybenzoic acid**	$C_7H_6O_3$	Benzene and substituted derivatives
**Abscisic acid**	$C_{15}H_{20}O_4$	Prenol lipids
**Amentoflavone**	$C_{30}H_{18}O_{10}$	Flavonoids
**Apigenin 4′, 7-O-diglucosideb 3**	$C_{27}H_{31}O_{15}$	Flavonoids (O-glycosides)
**Apigenin 7-neohesperidoside**	$C_{27}H_{30}O_{14}$	Flavonoids
**Apigenin 7-O-β-apiofuranosyl-6,8-di-C-β-glucopyranoside**	$C_{32}H_{38}O_{21}$	Flavonoids (O-glycosides)
**Apigenin O-rhamnosylglucoside**	$C_{27}H_{31}O_{14}$	Flavonoids (O-glycosides)
**Apigenin-6,8-di-C-β-glucopyranoside**	$C_{27}H_{30}O_{15}$	Flavonoids
**Apigenin-7-O-β-glucopyranoside**	$C_{21}H_{20}O_{10}$	Flavonoids (O-glycosides)
**Apiin**	$C_{26}H_{28}O_{14}$	Naphthalenes
**Azelaic acid**	$C_9H_{16}O_9$	Fatty Acyls

**Baicalein**	$C_{15}H_{10}O_5$	Flavonoids
**Calceolarioside**	$C_{23}H_{26}O_{11}$	Cinnamic acids and derivatives
**Chlorogenic acid**	$C_{16}H_{18}O_9$	Organooxygen compounds
**Chrysoeriol**	$C_{16}H_{12}O_6$	Flavonoids
**Chrysoeriol glucoside–xylosylglucoside malonylated (III)b 2**	$C_{36}H_{43}O_{23}$	Flavonoids
**Chrysoeriol O-glucoside**	$C_{22}H_{23}O_{11}$	Flavonoids (O-glycosides)
**Chrysoeriol O-glucoside malonylated (I)**	$C_{25}H_{25}O_{14}$	Flavonoids (O-glycosides)
**Chrysoeriol O-pentosylhexoside**	$C_{27}H_{30}O_{15}$	Flavonoids
**Cichoriin**	$C_{15}H_{16}O_{10}$	Coumarins
**Cinnamic acid glucoside**	$C_{15}H_{18}O_8$	Phenolic acids
**Dicaffeoylquinic acid**	$C_{25}H_{24}O_{12}$	Phenolic acids
**Eriodictyol**	$C_{15}H_{12}O_6$	Flavonoids
**Esculin**	$C_{15}H_{16}O_9$	Coumarins and derivatives
**Ferulic acid glucoside**	$C_{16}H_{20}O_9$	Organooxygen compounds
**Genistein**	$C_{15}H_{10}O_5$	Isoflavonoids
**Genistein 4′, 7-O-diglucoside dimalonylated (I) 2**	$C_{33}H_{35}O_{21}$	Isoflavonoids
**Genistein 6-C-glucoside 1**	$C_{21}H_{21}O_{10}$	Isoflavonoids
**Genistein 7-O-glucoside malonylated**	$C_{24}H_{23}O_{13}$	Isoflavonoids
**Genistein 8-C-glucosidee 2**	$C_{21}H_{21}O_{10}$	Isoflavonoids
**Genistein C-diglucoside 3**	$C_{27}H_{31}O_{15}$	Isoflavonoids
**Genistein C-diglucoside–xylosidec 3**	$C_{32}H_{39}O_{19}$	Isoflavonoids
**Genistein O-diglucoside malonylated (II) 2**	$C_{30}H_{33}O_{18}$	Isoflavonoids
**Isorhamnetin**	$C_{16}H_{12}O_7$	Flavonoids
**Kaempferol 3-O-glucoside**	$C_{21}H_{21}O_{11}$	Flavonoids
**Licodione**	$C_{15}H_{12}O_5$	Flavonoids
**Ligstroside**	$C_{25}H_{32}O_{12}$	Prenol lipids
**Ligstroside aglycone**	$C_{19}H_{22}O_7$	Prenol lipids
**Luteolin 7-O-glucoside**	$C_{21}H_{20}O_{11}$	Flavonoids
**Luteolin-4’-O-glucoside**	$C_{21}H_{20}O_{11}$	Flavonoids
**Luteone**	$C_{20}H_{18}O_6$	Flavonoids
**Naringenin**	$C_{15}H_{18}O_8$	Flavonoids
**p-coumaric acid glucoside**	$C_7H_6O_3$	Organooxygen compounds
**p-hydroxybenzoic acid**	$C_{15}H_{10}O_7$	Benzene and substituted derivatives
**Quercetin**	$C_{27}H_{31}O_{16}$	Flavonoids
**Quercetin rhamnosylglucoside 2**	$C_7H_{12}O_6$	Flavonoids
**Quinic acid**	$C_{15}H_{18}O_8$	Organooxygen

		compounds
**trans p-Coumaric acid**	$C_{27}H_{30}O_{15}$	Benzene and substituted derivatives
**Vicenin 2**	$C_{21}H_{20}O_{10}$	Flavonoids
**Vitexin**	$C_{20}H_{18}O_5$	Flavonoids
**Wighteone**	$C_{16}H_{16}O_5$	Flavonoids

Table S6. Suspect screening list for chickpea flour

Compound	Molecular formula	Class
**(Epi)afzelechin**	$C_{15}H_{14}O_5$	Flavonoids
**Apigenin 7-O-neohesperidoside**	$C_{27}H_{30}O_{14}$	Flavonoids
**Apigenin-6-C-glucoside**	$C_{21}H_{20}O_{10}$	Flavonoids
**Aromadendrin-3-O-β-D-glucopyranoside**	$C_{21}H_{22}O_{11}$	Flavonoids
**Benzoic acid**	$C_7H_6O_2$	Benzene and substituted derivatives
**Biochanin A 7-O-β-D-glucopyranoside**	$C_{22}H_{22}O_{10}$	Isoflavonoids
**Biochanin B**	$C_{16}H_{12}O_4$	Flavonoids
**Caffeoylquinic acid I**	$C_{16}H_{18}O_9$	Organooxygen compounds
**Caffeoylquinic acid I**	$C_{16}H_{18}O_9$	Organooxygen compounds
**Caffeoylquinic acid IV**	$C_{16}H_{18}O_9$	Organooxygen compounds
**Caffeoylquinic acid V**	$C_{16}H_{18}O_9$	Organooxygen compounds
**Cyanidin**	$C_{15}H_{11}O_6^+$	Flavonoids
**Daidzein**	$C_{15}H_{10}O_4$	Flavonoids
**Dalpanin I**	$C_{26}H_{30}O_{12}$	Lignan
**Delphinidin**	$C_{15}H_{11}ClO_7$	Anthocyanins
**Dihydrokaempferol [aromadendrin]**	$C_{15}H_{12}O_6$	Flavonoids
**Dihydroxybenzoic acid hexoside**	$C_{13}H_{16}O_9$	Benzene and substituted derivatives
**Dihydroxybenzoic acid hexoside deoxyhexoside**	$C_{19}H_{26}O_{13}$	Benzene and substituted derivatives
**Dihydroxybenzoic acid hexoside pentoside I**	$C_{18}H_{24}O_{13}$	Benzene and substituted derivatives
**Dihydroxybenzoic acid I**	$C_7H_6O_4$	Benzene and substituted derivatives
**Dihydroxybenzoic acid malonyl hexoside I**	$C_{16}H_{18}O_{12}$	Benzene and substituted derivatives
**Dihydroxybenzoic acid pentosided**	$C_{12}H_{14}O_8$	Benzene and substituted derivatives

**Ferulic acid hexoside**	$C_{16}H_{20}O_9$	Organooxygen compounds
**Gallic acid hexoside**	$C_{13}H_{16}O_{10}$	Benzene and substituted derivatives
**Genistein**	$C_{15}H_{10}O_5$	Isoflavonoids
**Hydroxybenzoic acid hexoside**	$C_{16}H_{16}O_8$	Phenolic acids
**Hydroxybenzoic acid hexoside pentoside I**	$C_{18}H_{24}O_{12}$	Benzene and substituted derivatives
**Isorhamentin 3-O-β-D-glucopyranoside**	$C_{22}H_{22}O_{12}$	Flavonoids
**Kaempferide**	$C_{16}H_{12}O_6$	Flavonoids
**Kaempferol 3,7-O-b-D-diglucopyranoside**	$C_{56}H_{100}O_6$	Flavonoids
**Kaempferol 3,7-O-β-D-diglucopyranoside**	$C_{27}H_{30}O_{16}$	Flavonoids
**Kaempferol 3-O-lathyroside-7-O-α-L-rhamnopyranoside**	$C_{32}H_{38}O_{19}$	Flavonoids
**Kaempferol 3-O-rutinoseb**	$C_{27}H_{30}O_{15}$	Flavonoids
**Kaempferol 3-O-β-D-diglucopyranoside**	$C_{27}H_{30}O_{16}$	Flavonoids
**Kaempferol 3-O-β-d-glucopyarnoside**	$C_{21}H_{20}O_{11}$	Flavonoids
**Kaempferol malonyl dihexoside I**	$C_{30}H_{32}O_{19}$	Flavonoids
**Kaempferol malonyl dihexoside pentoside I**	$C_{35}H_{40}O_{23}$	Flavonoids
**Kaempferol-3,4′-O-β-D-diglucopyranoside**	$C_{27}H_{30}O_{16}$	Flavonoids
**Kaempferol-3-O-(6″-malonyl-)-β-D-glucopyranoside**	$C_{24}H_{22}O_{14}$	Flavonoids
**Kaempferol-3-O-rutinoside-7-O-β-D-glucopyranoside**	$C_{33}H_{40}O_{20}$	Flavonoids
**Kaempferol-3-O-β-D-glucopyranoside-7-O-α-l-rhamnopyranside**	$C_{27}H_{30}O_{15}$	Flavonoids
**Luteolin-3,7-di-O-glucoside**	$C_{27}H_{30}O_{16}$	Flavonoids
**Malvidin**	$C_{17}H_{15}O_7^+$	Flavonoids
**Methoxy hydroxybenzoic acid hexoside pentosidee**	$C_{19}H_{26}O_{12}$	Benzene and substituted derivatives
**Myricetin-3-O-rhamnoside**	$C_{21}H_{20}O_{12}$	Flavonoids
**Myricetin-O-methyl ether hexoside deoxyhexoside**	$C_{28}H_{32}O_{17}$	Flavonoids
**Myricetin-O-methyl ether hexoside deoxyhexoside pentoside**	$C_{33}H_{40}O_{21}$	Flavonoids
**Naringenin dihexoside pentoside**	$C_{32}H_{40}O_{19}$	Flavonoids
**Naringenin hexoside pentoside I**	$C_{26}H_{30}O_{14}$	Flavonoids
**Orobol**	$C_{15}H_{10}O_6$	Isoflavonoids
**p-Coumaric acid glucopyranoside**	$C_{15}H_{18}O_8$	Organooxygen compounds
**Petunidin**	$C_{16}H_{13}O_7^+$	Anthocyanins
**p-hydroxybenzoic acid**	$C_7H_6O_3$	Benzene and substituted derivatives
**Pratensein**	$C_{16}H_{12}O_6$	Flavonoids

**Pratensein 7-O-β-D-glucopyranoside**	$C_{22}H_{22}O_{11}$	Isoflavonoids
**Prunin [naringenin 7-O-β-D-glucopyranoside]**	$C_{21}H_{22}O_{10}$	Flavonoids
**Quercetin 3-O-(6″-malonylneohesperidoside)**	$C_{30}H_{32}O_{19}$	Flavonoids
**Quercetin 3-O-β-d-glucopyranosideb**	$C_{21}H_{20}O_{12}$	Flavonoids
**Quercetin-3,7-O-di-glucopyranoside**	$C_{27}H_{30}O_{17}$	Flavonoids
**Quercetin-3-O-galactoside**	$C_{21}H_{20}O_{12}$	Flavonoids
**Quercetin-3-O-rhamnoside**	$C_{21}H_{20}O_{11}$	Flavonoids
**Quercetin-3-O-rutinoside-7-O-α-L-rhamnopyranoside**	$C_{33}H_{40}O_{20}$	Flavonoids
**Quercetin-3-O-β-D-glucopyranuronic acid**	$C_{21}H_{18}O_{13}$	Flavonoids
**Sinapic acid hexoside I**	$C_{17}H_{22}O_{10}$	Hydroxycinammic acids
**Vanillic acid hexoside pentoside**	$C_{19}H_{26}O_{13}$	Benzene and substituted derivatives
**Vanillic acid hexoside pentoside I**	$C_{19}H_{26}O_{13}$	Benzene and substituted derivatives
**Vanillic acid-4-O-b-D-glucopyranoside**	$C_{14}H_{18}O_9$	Benzene and substituted derivatives

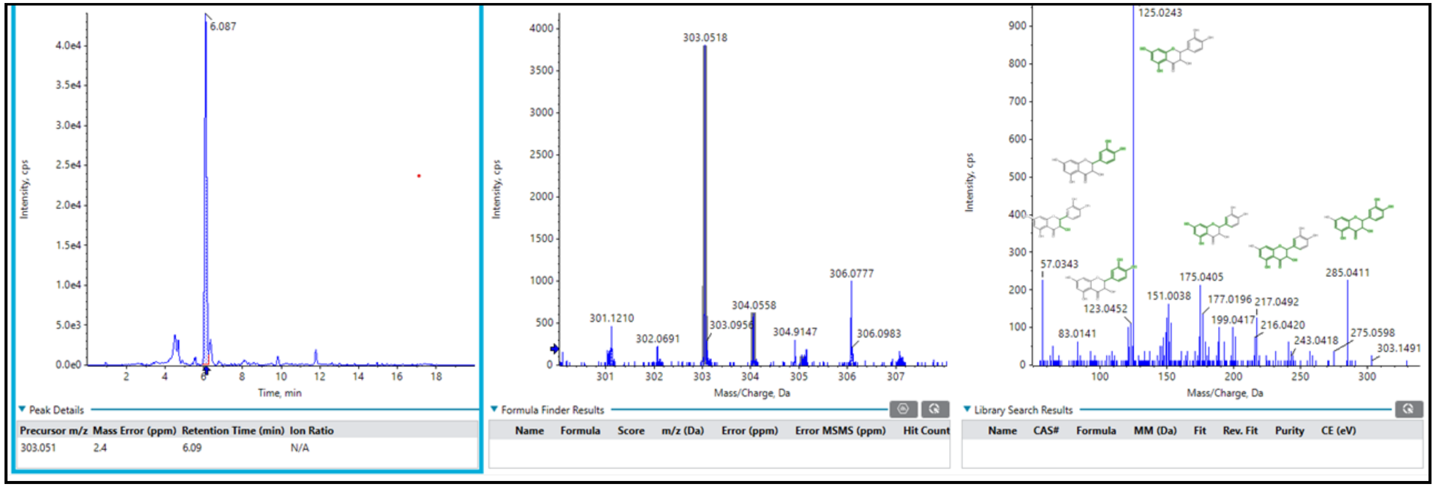

Figure S1. Extracted ion chromatogram, MS, and MS/MS spectra of taxifolin in LFL.

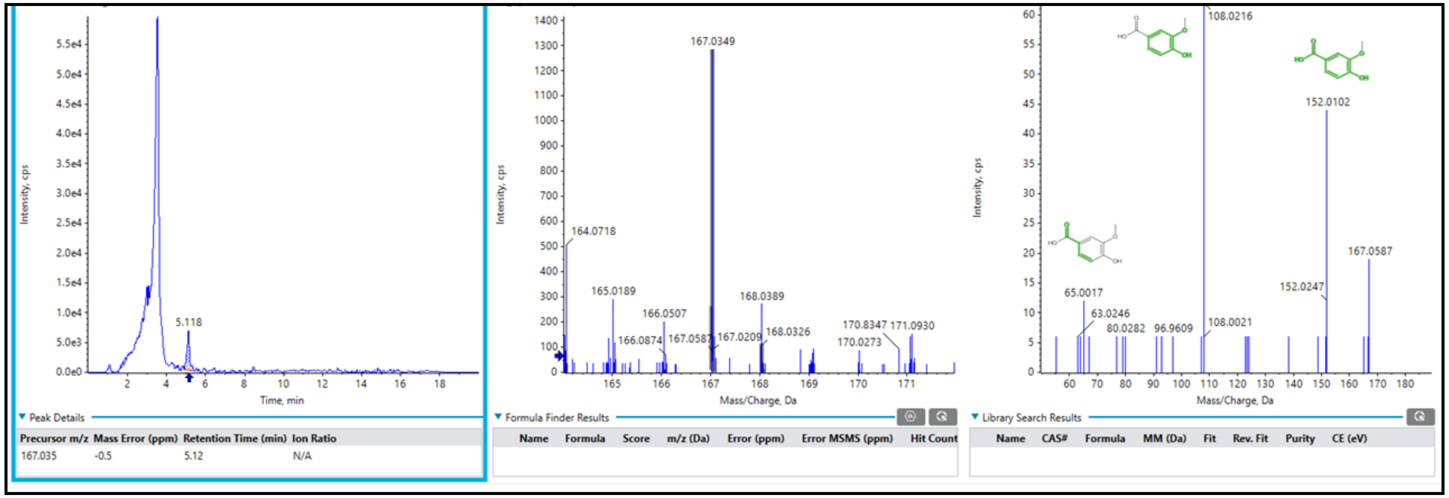

Figure S2. Extracted ion chromatogram, MS, and MS/MS spectra of vanillic acid in LFC.

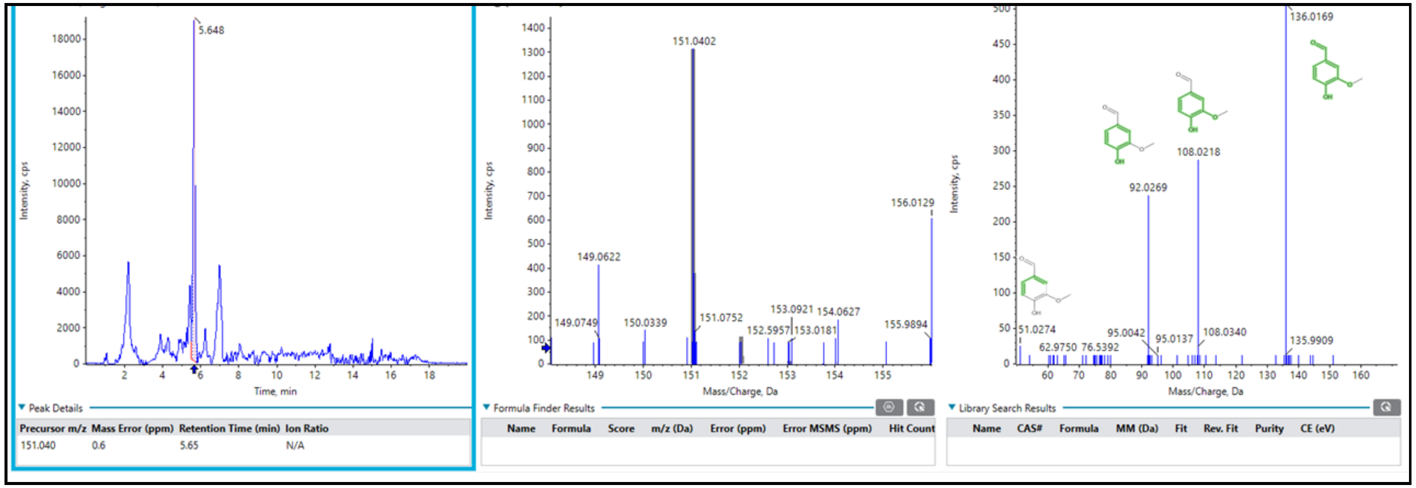

Figure S3. Extracted ion chromatogram, MS, and MS/MS spectra of vanillin in LFL.

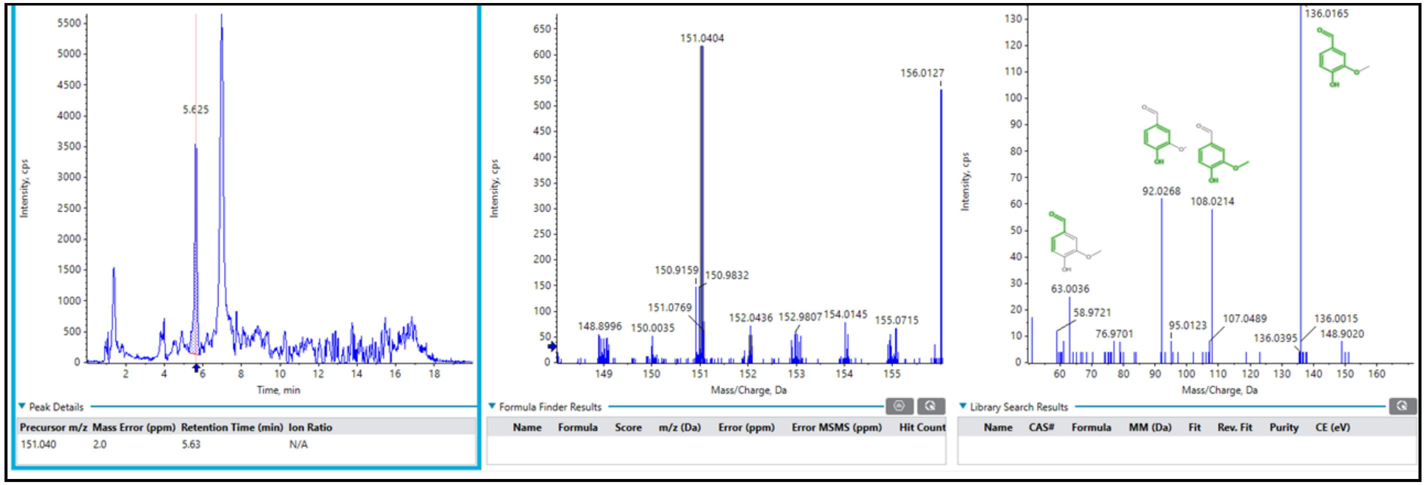

Figure S4. Extracted ion chromatogram, MS, and MS/MS spectra of vanillin in WFL.

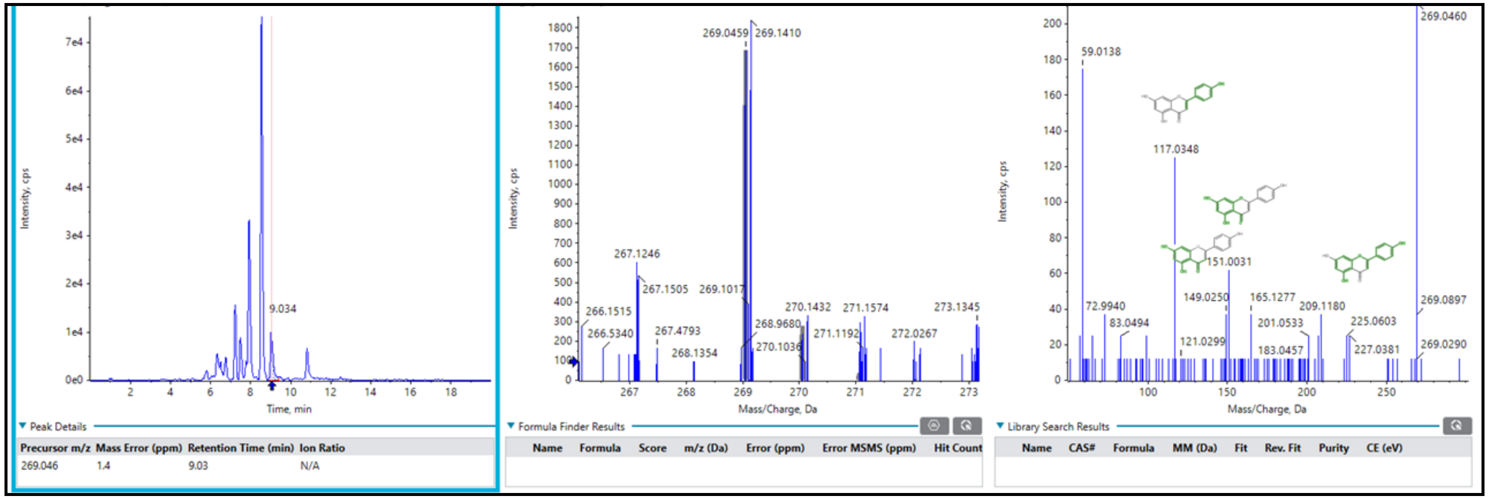

Figure S5. Extracted ion chromatogram, MS, and MS/MS spectra of apigenin in LFL.

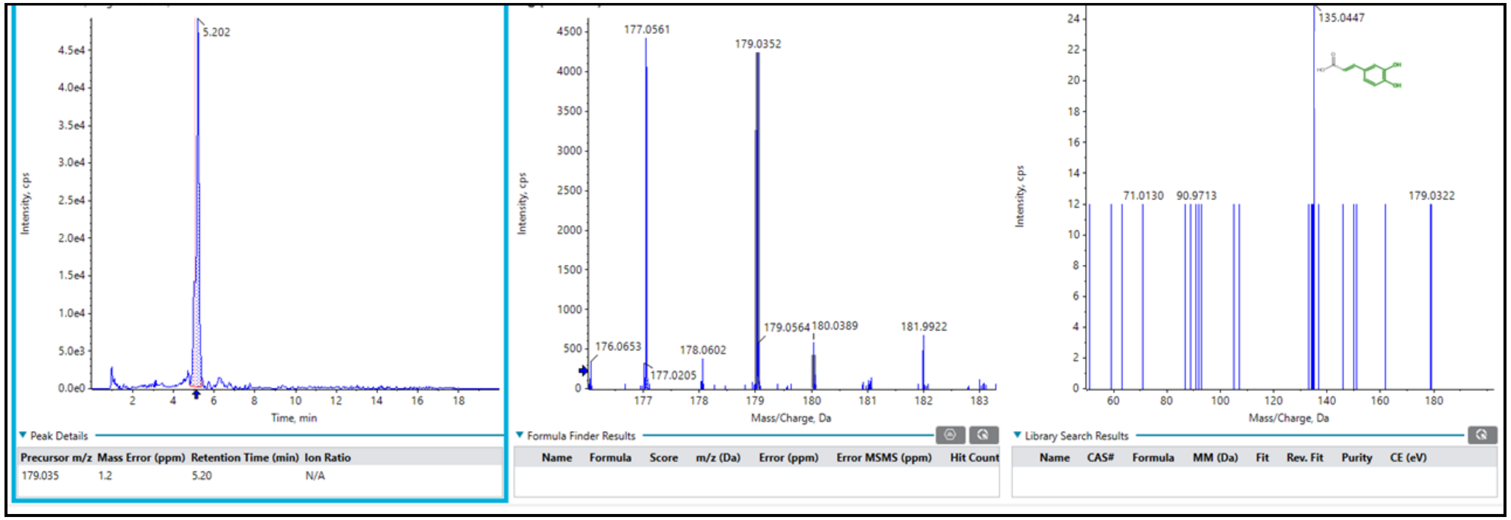

Figure S6. Extracted ion chromatogram, MS, and MS/MS spectra of caffeic acid in LFL.

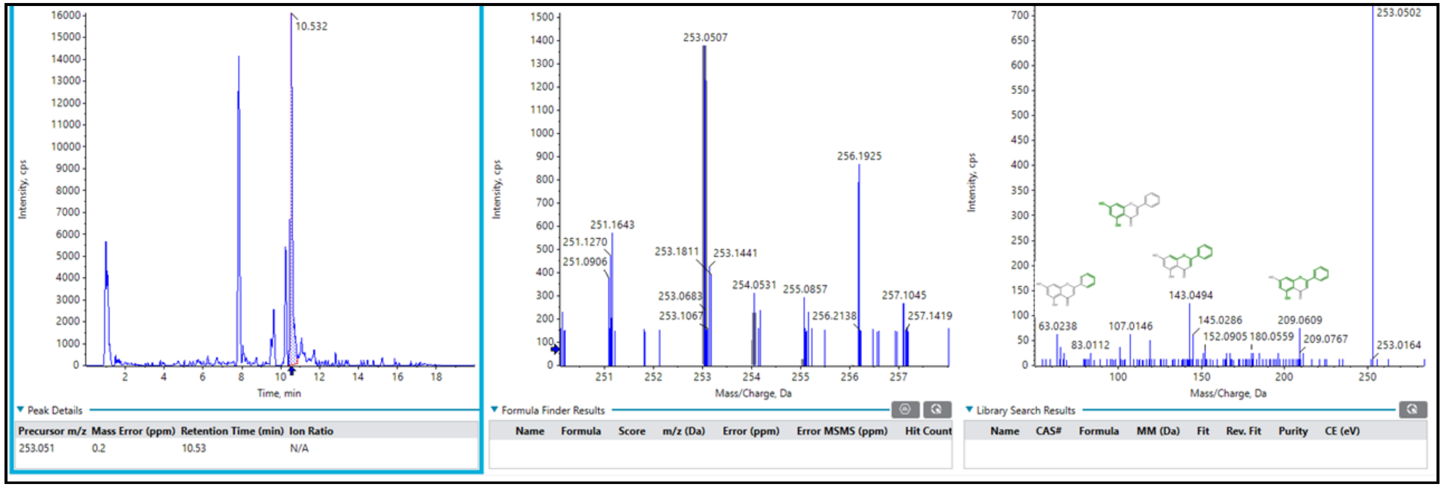

Figure S7. Extracted ion chromatogram, MS, and MS/MS spectra of chrysin in CFL.

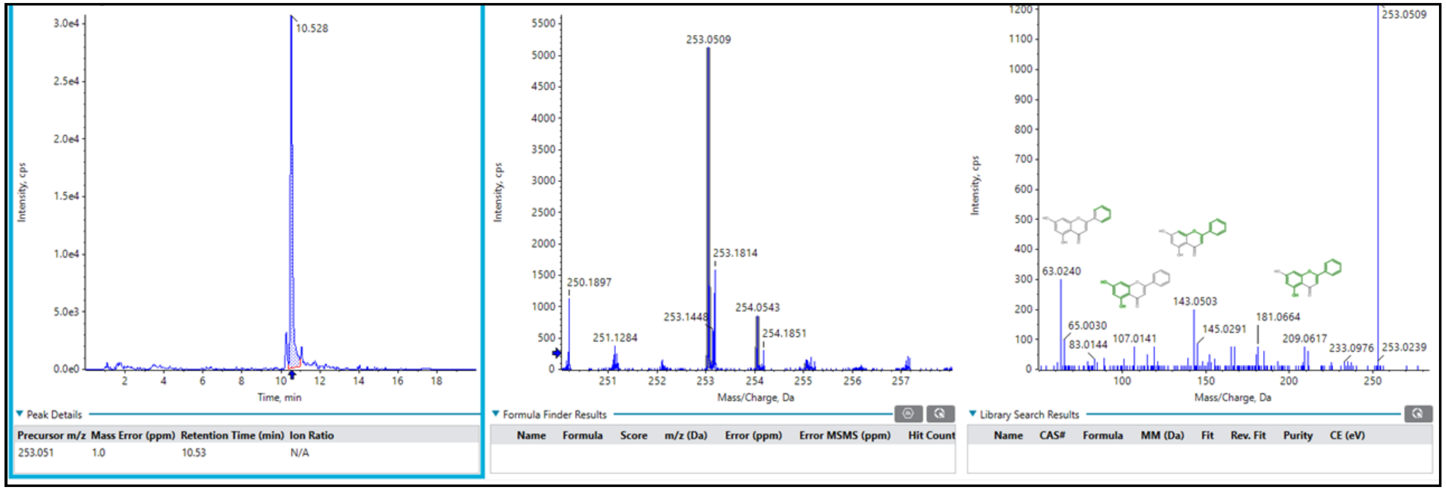

Figure S8. Extracted ion chromatogram, MS, and MS/MS spectra of chrysin in LFC.

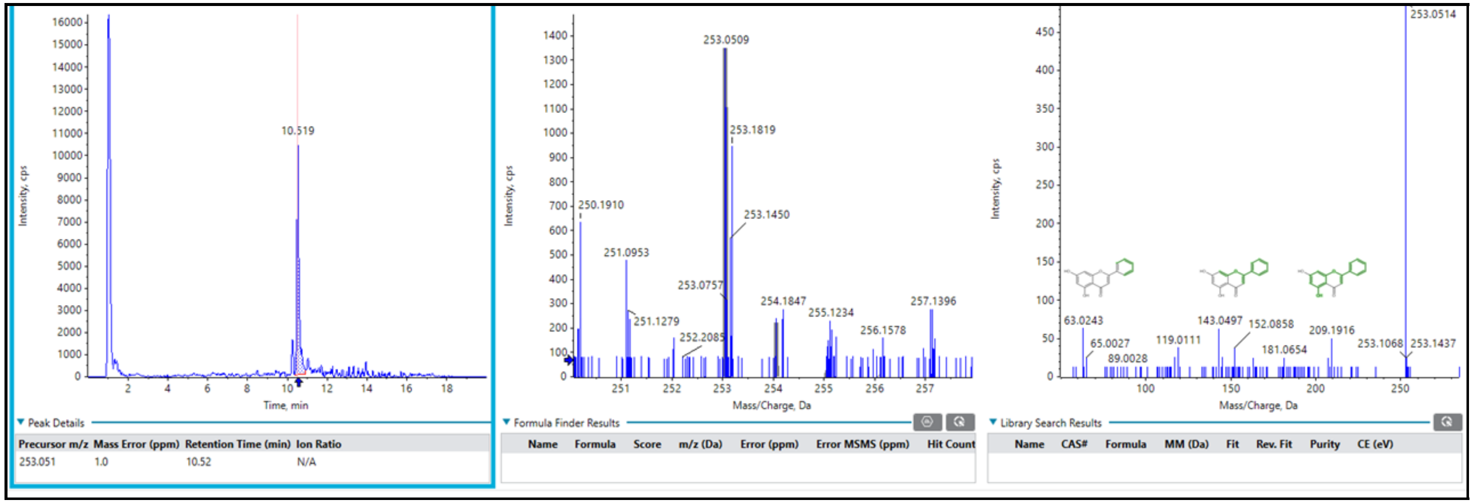

Figure S9. Extracted ion chromatogram, MS, and MS/MS spectra of chrysin in WFL.

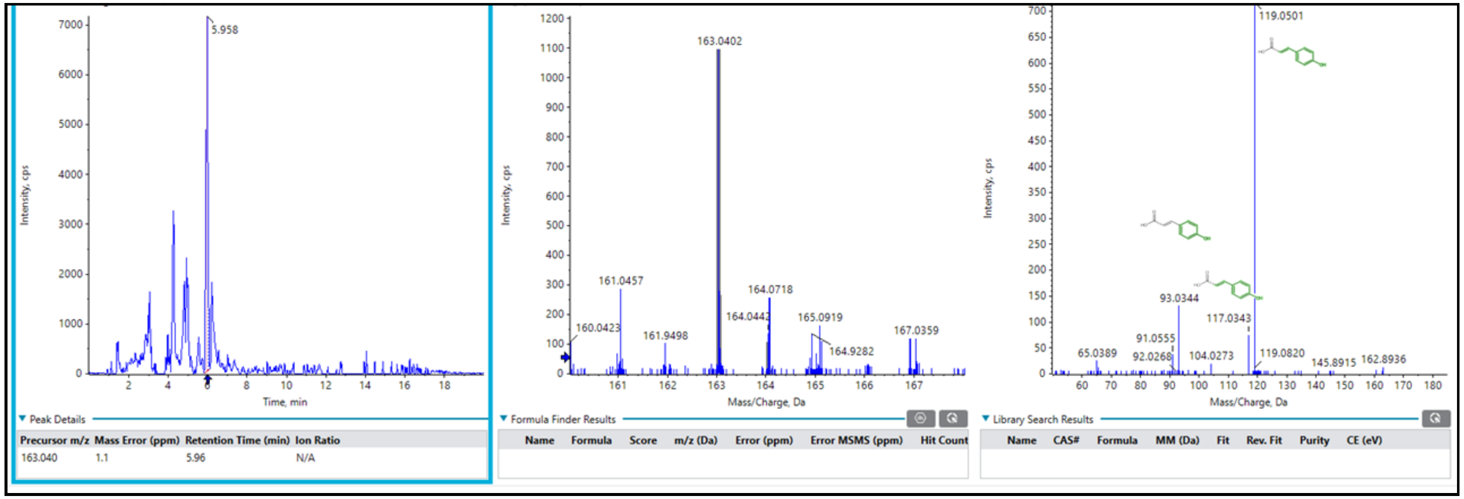

Figure S10. Extracted ion chromatogram, MS, and MS/MS spectra of coumaric acid in CFL.

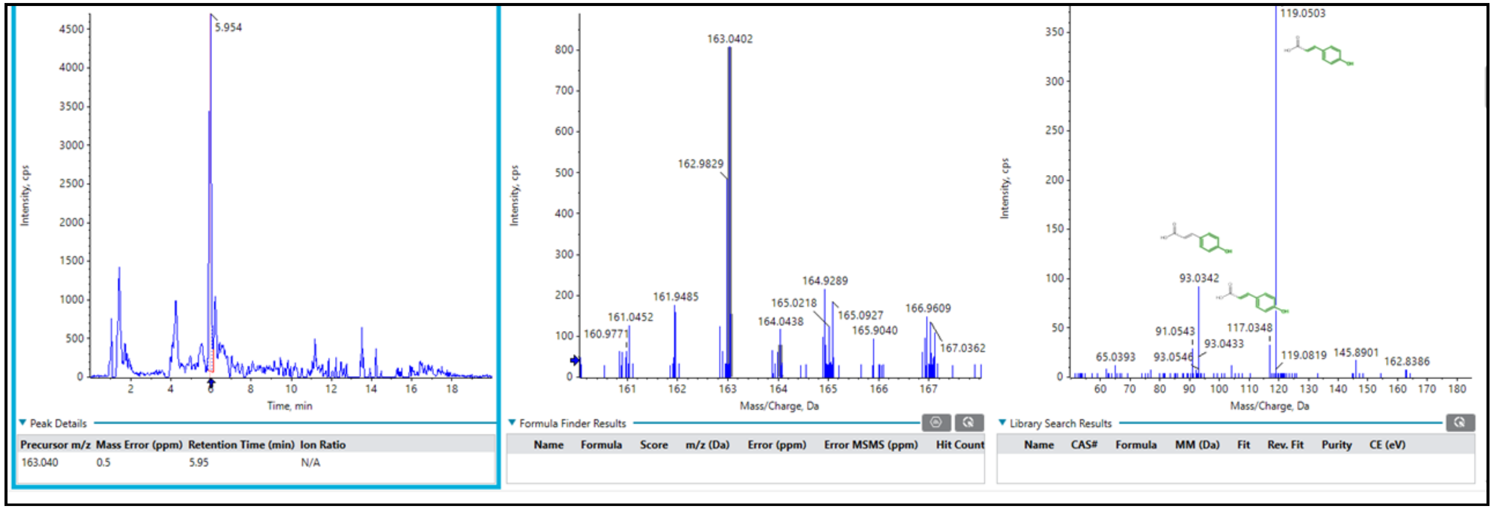

Figure S11. Extracted ion chromatogram, MS, and MS/MS spectra coumaric acid in WFL.

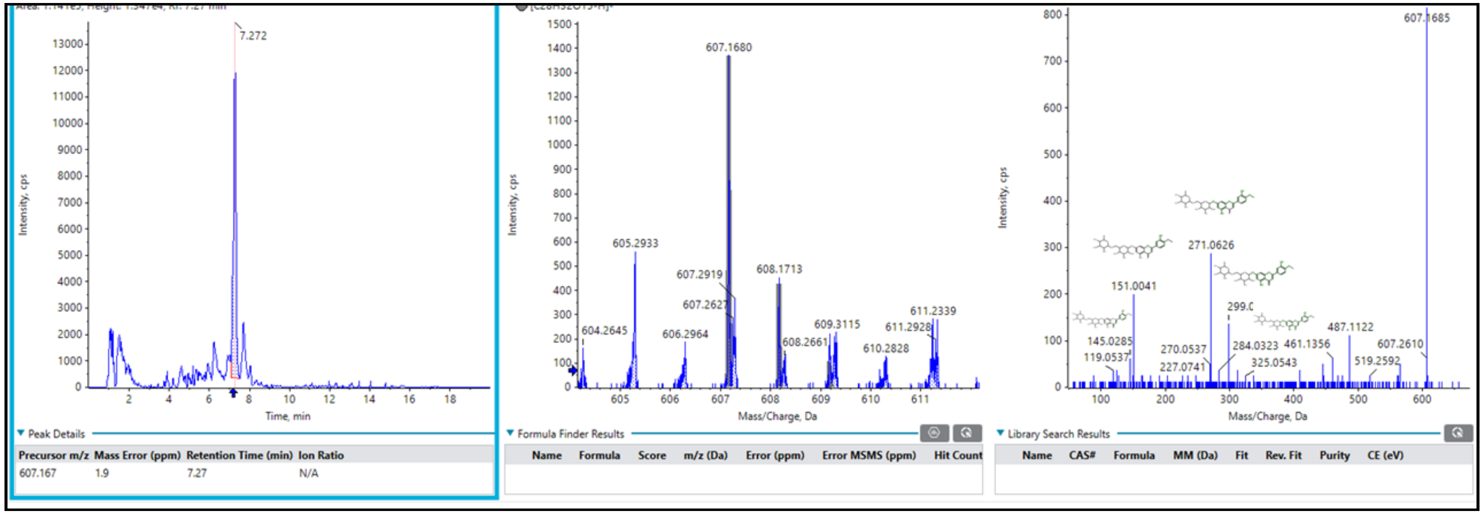

Figure S12. Extracted ion chromatogram, MS, and MS/MS spectra of diosmin in CFL.

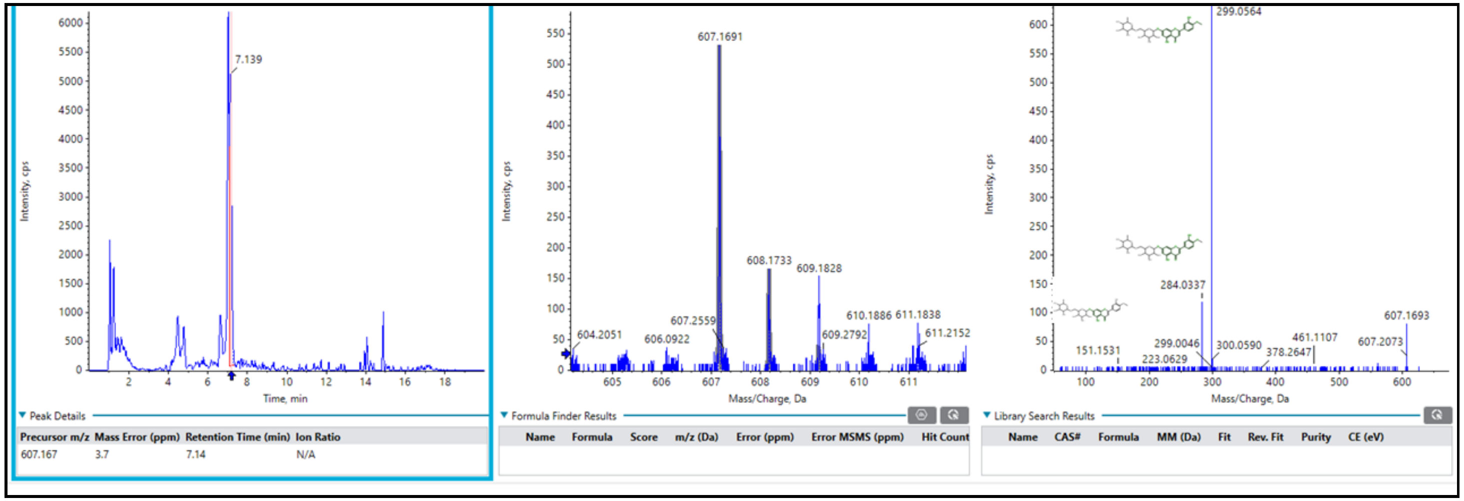

Figure S13. Extracted ion chromatogram, MS, and MS/MS spectra of diosmin in WFL.

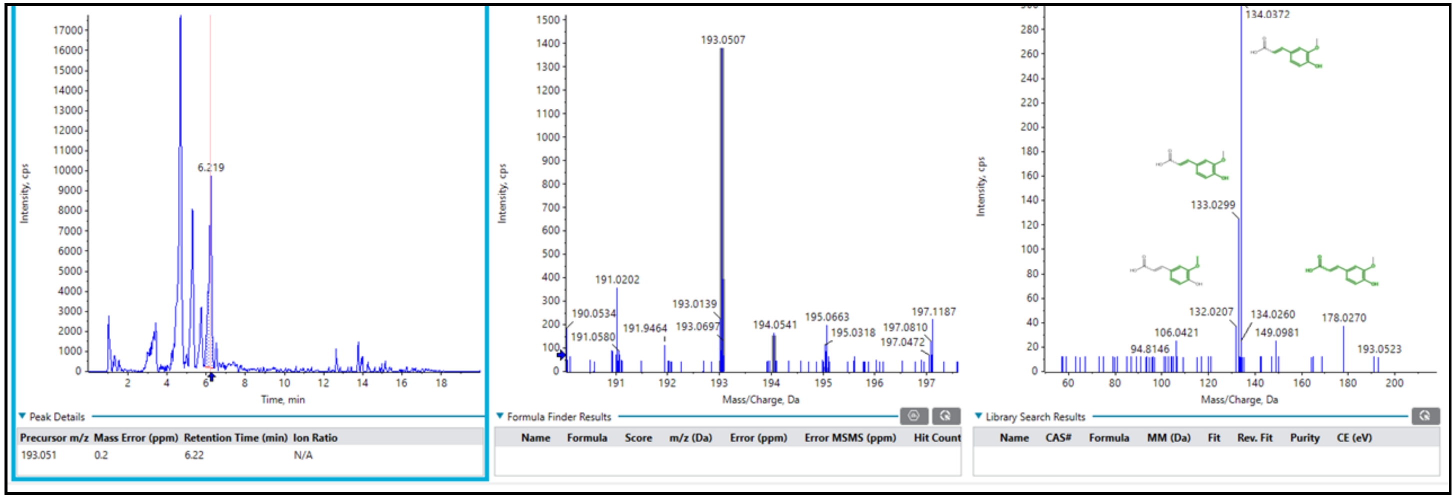

Figure S14. Extracted ion chromatogram, MS, and MS/MS spectra of ferulic acid in CFL.

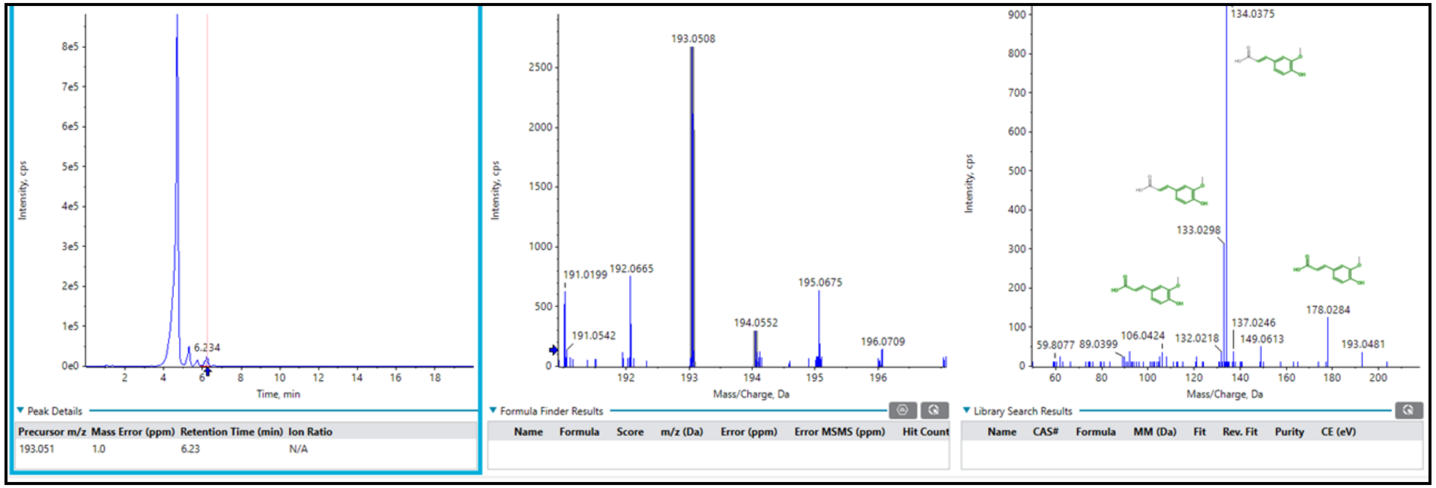

Figure S15. Extracted ion chromatogram, MS, and MS/MS spectra of ferulic acid in LFC.

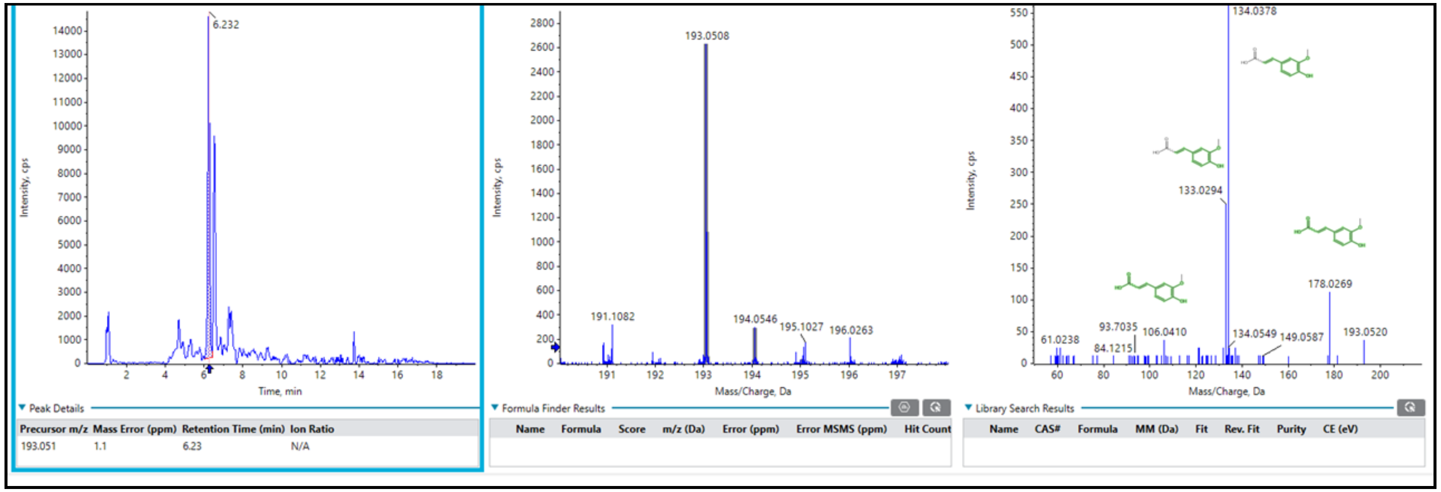

Figure S16. Extracted ion chromatogram, MS, and MS/MS spectra of ferulic acid in WFL.

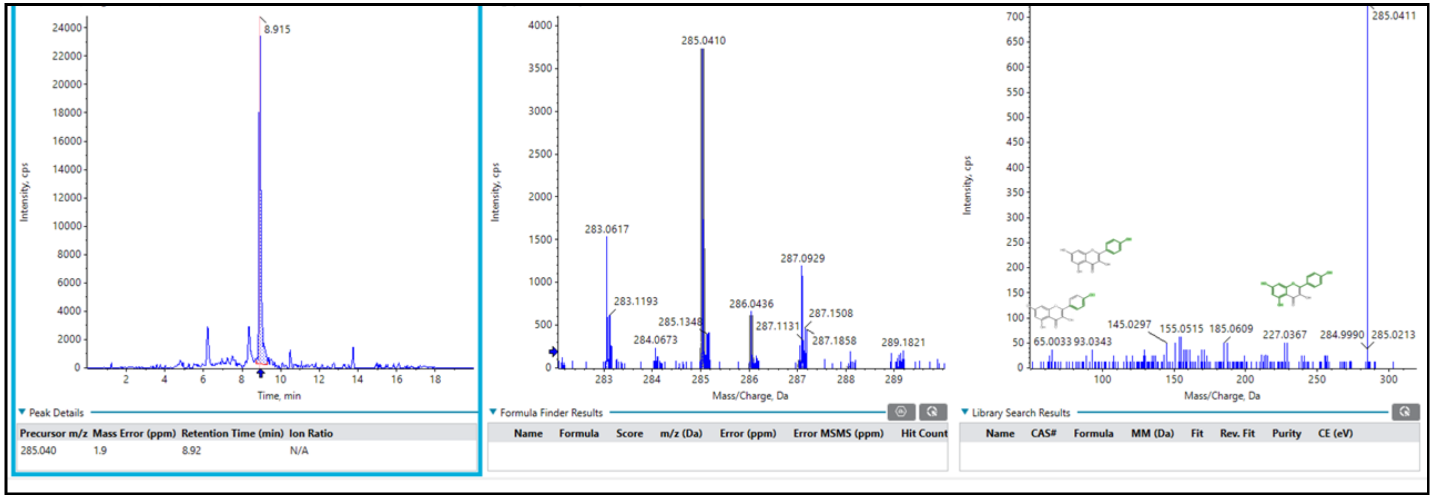

Figure S17. Extracted ion chromatogram, MS, and MS/MS spectra of kaempferol in CFL.

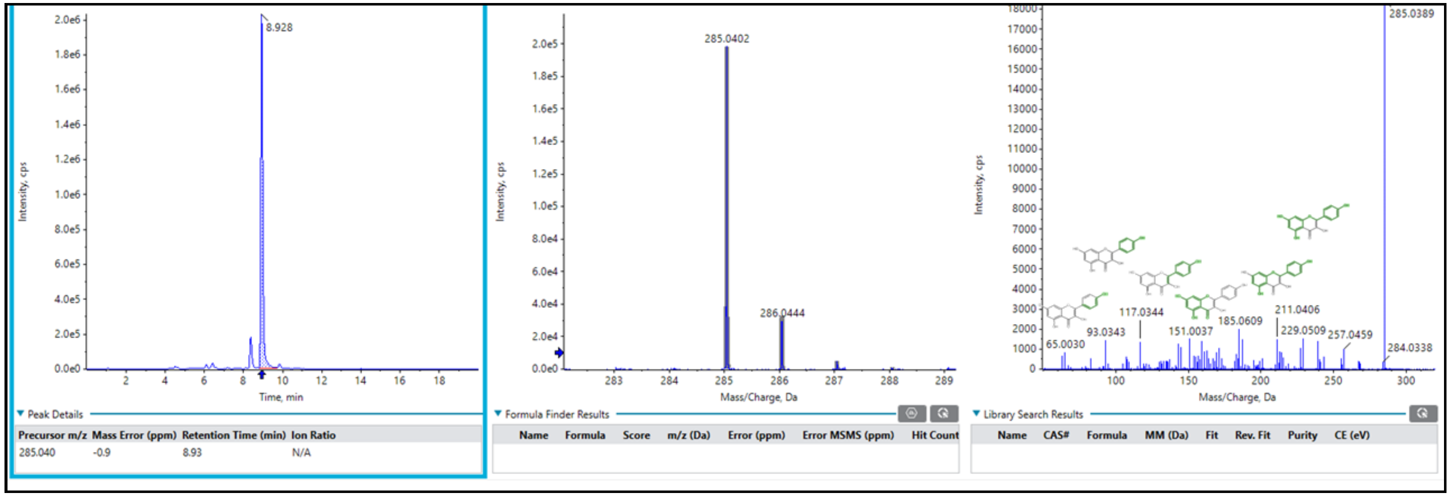

Figure S18. Extracted ion chromatogram, MS, and MS/MS spectra of kaempferol in CFC.

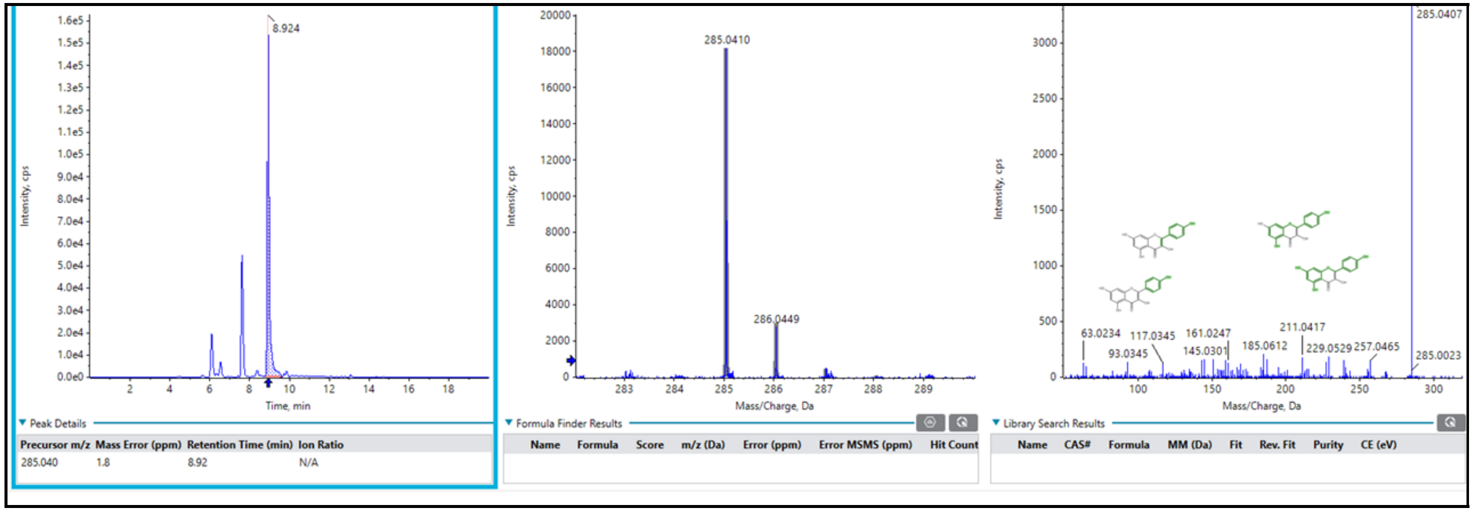

Figure S19. Extracted ion chromatogram, MS, and MS/MS spectra of kaempferol in LFL.

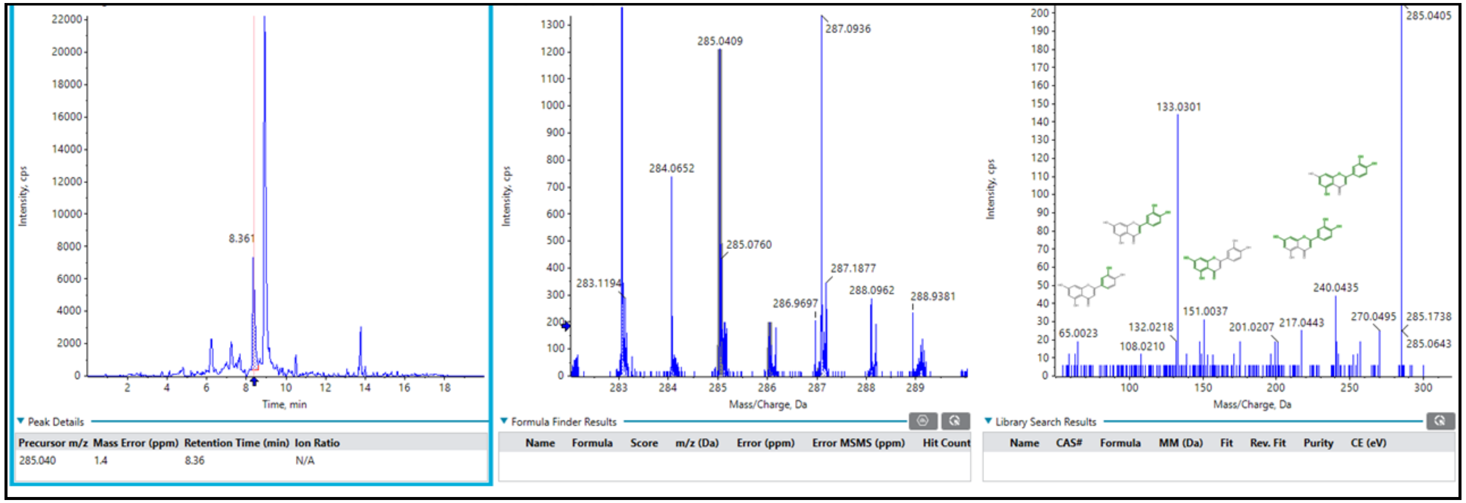

Figure S20. Extracted ion chromatogram, MS, and MS/MS spectra of luteolin in CFL.

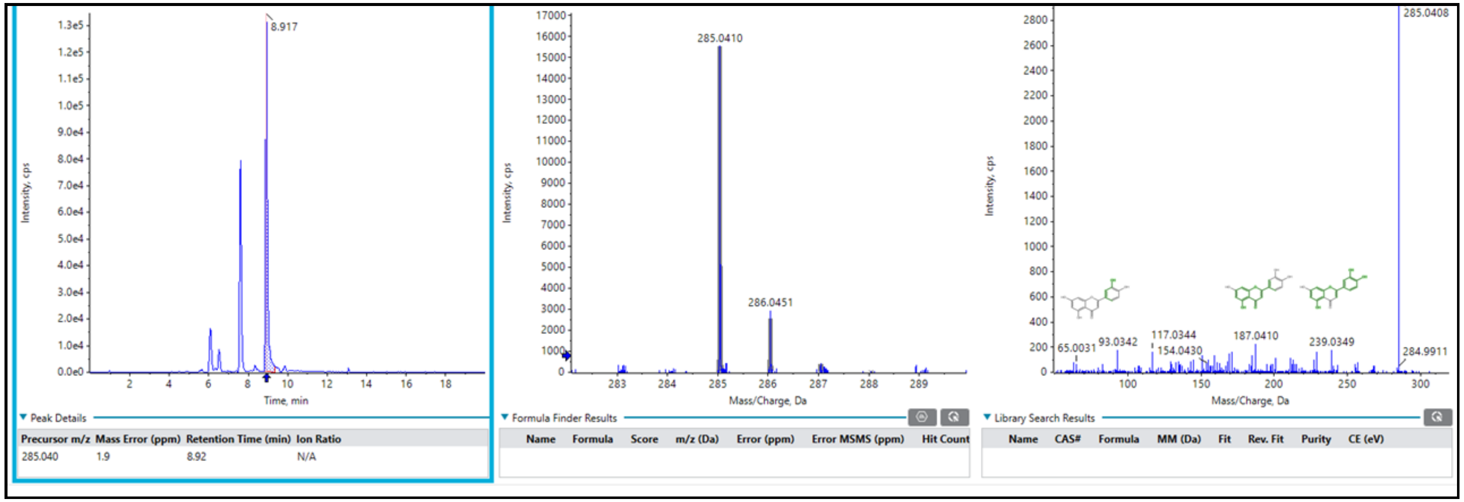

Figure S21. Extracted ion chromatogram, MS, and MS/MS spectra of luteolin in LFL.

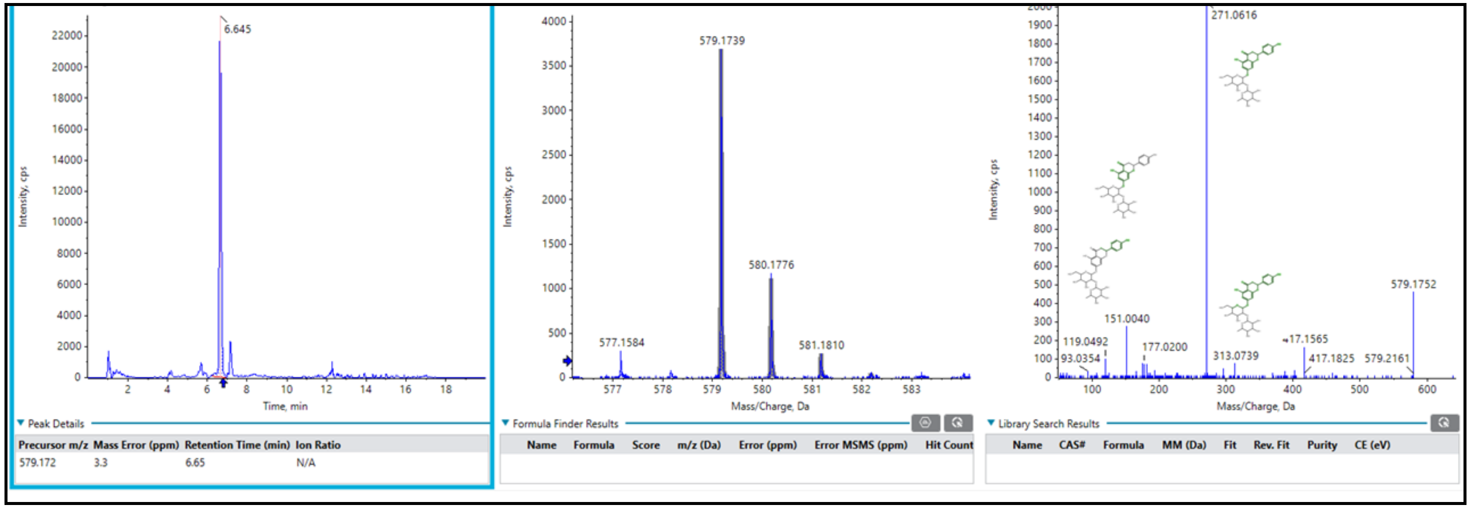

Figure S22. Extracted ion chromatogram, MS, and MS/MS spectra of naringin in WFC.

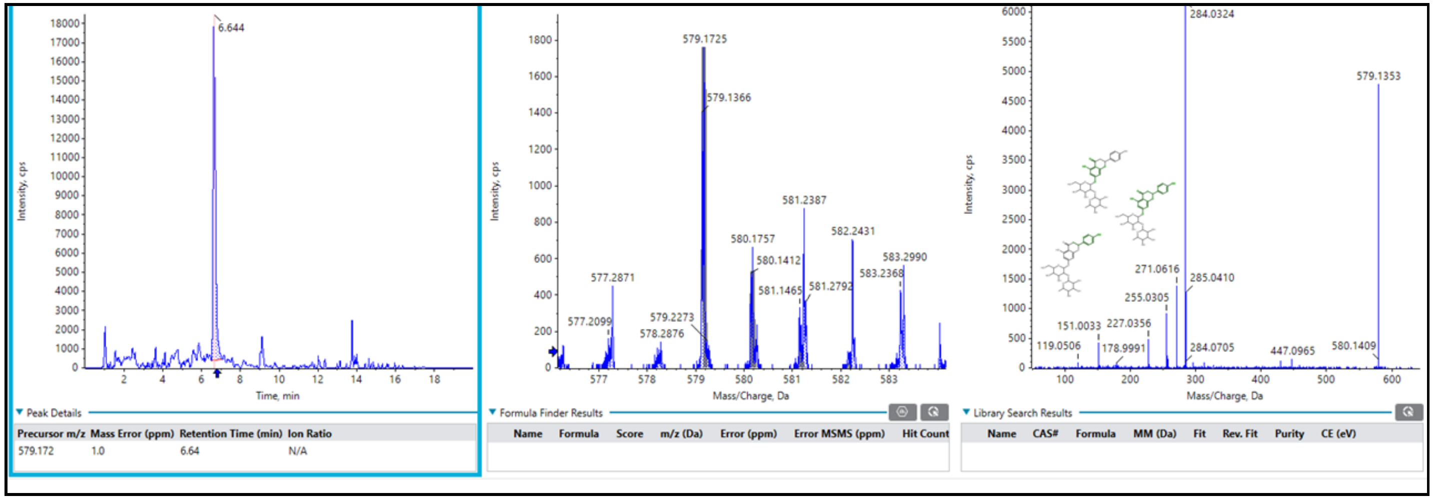

Figure S23. Extracted ion chromatogram, MS, and MS/MS spectra of naringin in CFL.

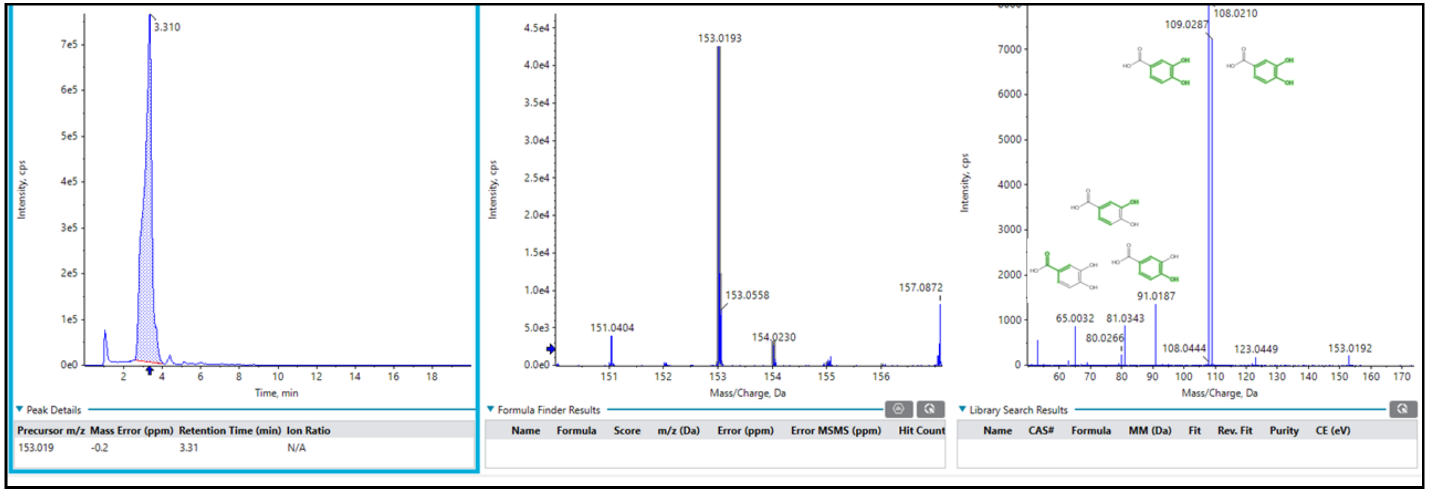

Figure S24. Extracted ion chromatogram, MS, and MS/MS spectra of procatechuic acid in CFL.

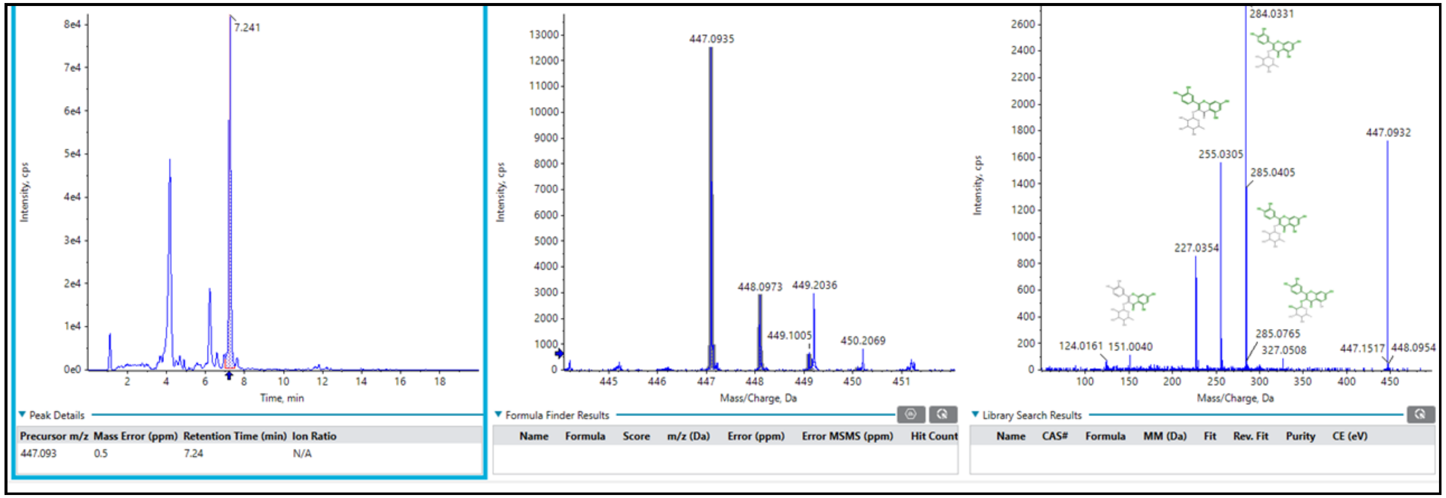

Figure S25. Extracted ion chromatogram, MS, and MS/MS spectra of quercitrin in CFL.

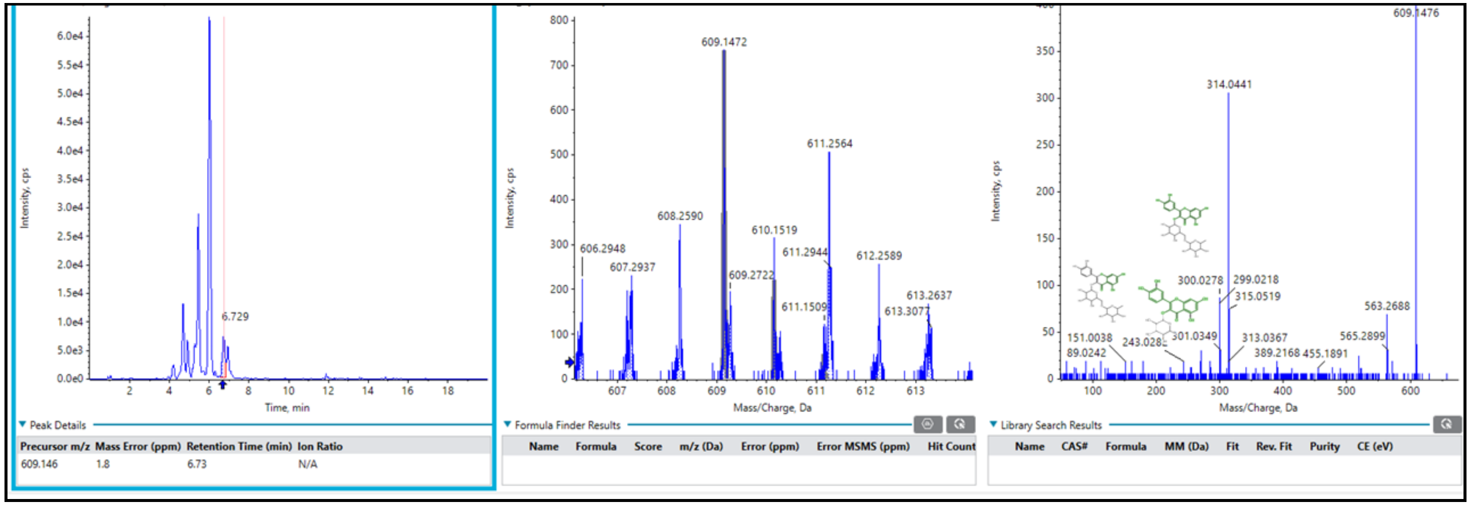

Figure S26. Extracted ion chromatogram, MS, and MS/MS spectra of rutin in CFL.

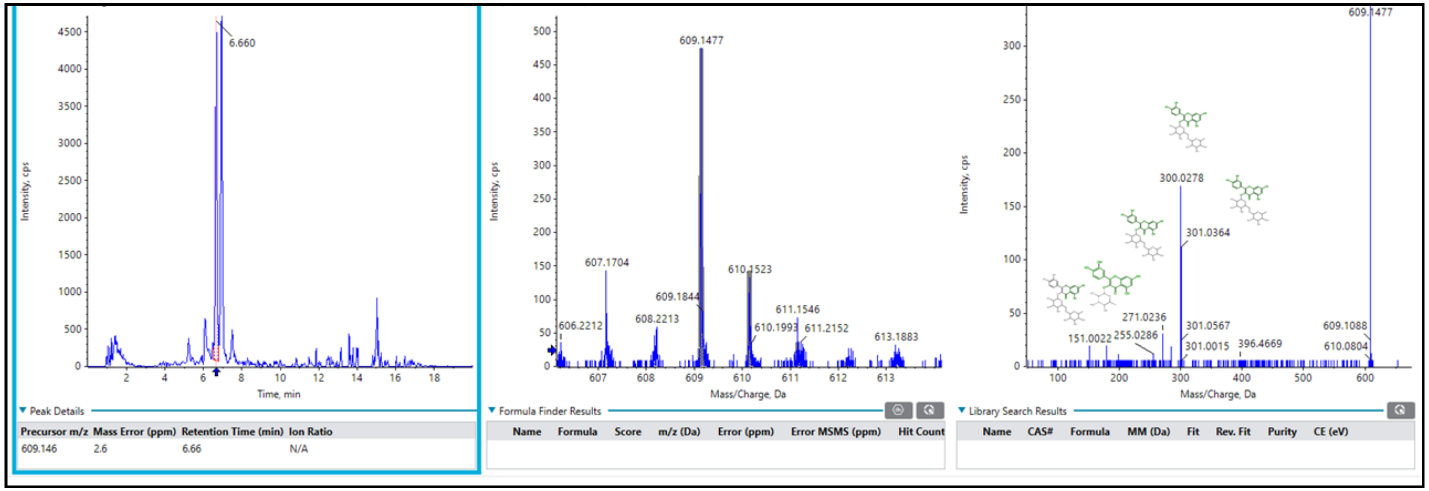

Figure S27. Extracted ion chromatogram, MS, and MS/MS spectra of rutin in WFC
